# *In silico* and *in vitro* assessment of an optimized QbD-guided myoinositol and metformin-loaded mucus-penetrating particle-based gel for the amelioration of PCOS†

**DOI:** 10.1039/d3na00943b

**Published:** 2024-01-03

**Authors:** Uzma Farooq, Mohd Aamir Mirza, Abdullah Alshetaili, Sradhanjali Mohapatra, Pooja Jain, Nazia Hassan, Zeenat Iqbal, Asgar Ali

**Affiliations:** a Department of Pharmaceutics, School of Pharmaceutical Education and Research (SPER), Jamia Hamdard New Delhi 110062 India aali@jamiahamdard.ac.in zeenatiqbal@jamiahamdard.ac.in +91-9899571726 +91-9811733016; b Department of Pharmaceutics, College of Pharmacy, Prince Sattam Bin Abdulaziz University Alkharj Saudi Arabia

## Abstract

Polycystic ovarian syndrome (PCOS) is a multi-factorial endocrine disorder affecting women of reproductive age. However, its high prevalence and the unsuccessful translation of conventional modalities have made PCOS a pharmaco-therapeutic challenge. In the present study, we explored bi-formulations (comprising metformin-loaded mucus-penetrating nanoparticles, MTF-MPPs, and myoinositol-loaded mucus-penetrating particles, MI-MPPs) incorporated in a carbomer gel tailored for intravaginal administration. For the development and optimization of the MPPs-gel, a QbD (quality by design) approach was employed, including the initial and final risk assessment, central composite design of experts, and method validation. The optimized MTF-MPPs and MI-MPPs possessed an optimum nanometric particle size (195.0 nm and 178.8 nm, respectively) and a PDI of 0.150 and 0.123, respectively, together with a negligible negative zeta potential (−5.19 mV and −6.19 mV, respectively) through the vaginal mucus. It was observed that the MPPs are small and monodisperse with a neutral surface charge. It was observed that the MPPs-gel formulations released approximately 69.86 ± 4.65% of MTF and 67.14 ± 5.74% of MI within 120 h (5 days), which was observed to be sustained unlike MFT-MI-gel with approximately 94.89 ± 4.17% of MTF and 90.91 ± 15% of MI drugs released within 12 h. The confocal microscopy study of rhodamine-loaded MPPs indicated that they possessed a high fluorescence intensity at a depth of 15 μm, while as the penetration trajectory in the vaginal tissue increased to 35 μm, their intensity was reduced, appearing to be more prominent in the blood vessels. The analyzed data of MPPs-gel suggest that the optimized MPPs-gel formulation has potential to reach the targeted area *via* the uterovaginal mucosa, which has a wide network of blood vessels. Subsequently, *in vivo* studies were conducted and the results revealed that the proposed MPPs-gel formulation could regulate the estrous cycle of the reproductive system compared to the conventional formulation. Moreover, the formulation significantly reduced the weight of the ovaries compared to the control and conventional vaginal gel. Biochemical estimation showed improved insulin and sex hormone levels. Thus, the obtained data revealed that the deep penetration and deposition of MTF and MI on the targeted area through intravaginal delivery resulted in better therapeutic effects than the conventional vaginal gel. The obtained results confirmed the amelioration of PCOS upon treatment using the prepared MPPs-gel formulation. According to the relevant evaluation studies, it was concluded that MPPs-gel was retained in the vaginal cavity for systemic effects. Also, the sustained and non-irritating therapeutic effect meets the safety aspects. This work serves as a promising strategy for intravaginal drug delivery.

## Abstract 2

Mechanistic process development and effect on the polycystic ovarian syndrome (PCOS).

## Graphical abstract

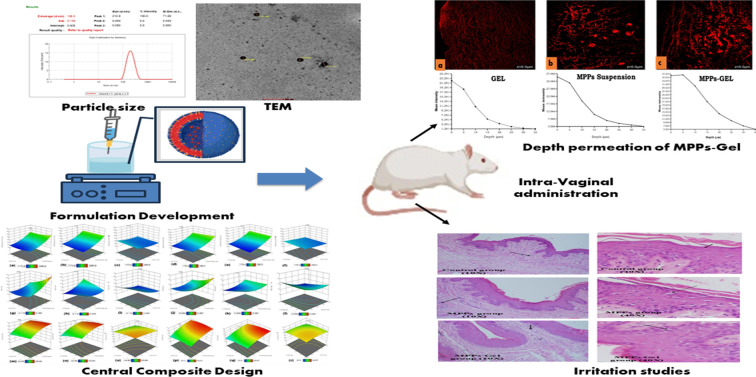

## 1. Introduction

PCOS is a multifactorial, endocrinopathy commonly occurring in women of reproductive age. The prevalence of PCOS is alarming, with an estimated global dominance of 20%. Furthermore, the challenges of social stigma, ineffective conventional modalities, associated co-morbidities (insulin resistance, obesity, and infertility), and compromised quality of life (QoL) have made PCOS a burgeoning pharmaco-therapeutic challenge. The clinical diagnosis of PCOS involves two of any three factors: hyperandrogenism, ovarian dysfunction, and formation of cysts in the ovaries. The major associated complications of PCOS are irregular menstrual cycle, insulin resistance, diabetes, obesity, anovulation, and infertility. The pathogenesis of insulin resistance involves defects in the insulin signalling pathway (inositol-containing phosphoglycan mediators).^1,2^

Although insulin resistance (IR) does not directly influence the diagnostic criteria of PCOS, it plays a key role in its pathogenesis (70–80% prevalence of IR has been reported).^3^ The normal physiological mechanism of a healthy woman's ovary is comprised of a 99% intracellular pool of myoinositol (MI), while the remaining 1% is comprised of d-chiro-inositol (DCI). MI is a key factor in regulating the glucose uptake in the ovaries and FSH signaling, which is essential for the meiosis in follicles. DCI is used to control the insulin-mediated synthesis of androgen. However, women with PCOS reportedly exhibit a disrupted inherent ratio of inositol isomers, which is due to MI deficiency, ultimately compromising follicle-stimulating hormone (FSH) signaling.^4,5^

Accordingly, an extrinsic supply of MI seems pertinent. Therefore, in this study, we focused on combining metformin (MTF) and myoinositol (MI) in a single system. MI is used as a nutritional supplement to alleviate the symptoms of PCOS, while MTF is used to treat irregular menstrual cycles in PCOS. The solid, synergistic, and complementary activities of both drugs are envisage to alleviate ovarian cysts and restore hormonal balance, both of which are imperative for the amelioration of PCOS.^6^

Gynaecological treatment with a conventional oral drug delivery system is highly recommended as an adjuvant therapy for PCOS amelioration. However, the oral route is associated with several problems such as high dose frequency, high risk of toxicity, first-pass metabolism, bioavailability issue, drug absorption problems, and related side effects. Furthermore, the therapeutic efficacy of pharmacologically active compounds administered by the conventional route is limited by several biological barriers. Moreover, complete systemic administration of therapeutics leads to off-target adverse effects, where most of the drugs may be metabolized and inactivated. Additionally, other conventional routes such as the intravenous (IV) route bypass the barrier of the gastrointestinal tract, but due to the labyrinthine blood vessels in the human, high doses must be used to maintain the therapeutic level of drugs in a particular location, which may lead to off-target adverse effects and toxicity. Furthermore, it also achieves rapid clearance, making a high frequency of dosing necessary. In this case, the advanced design of pharmaceutical formulations and the appropriate route of administration have the potential to overcome these challenges and improve patient compliance and therapeutic efficacy.

The vaginal lumen has also been explored as a suitable route of drug delivery to avoid systemic effects. Due to the presence of a wide network of blood vessels in the vagina, it is a promising excellent route for systemic and local drug delivery. The vaginal route of drug delivery facilitates the use of prolonged dosing regimens, continuous release of drugs, and low dosing frequency. Also, the intravaginal route seems promising for sustained and targeted drug delivery; however, the mucosal lining poses an inherent barrier.^7^ Specifically, the luminal vaginal mucus layer traps foreign material or conventional pharmaceuticals by bio-adhesion and steric hindrance. The trapped particles are rapidly cleared following a self-cleansing mechanism in the vaginal cavity.^8^ Thus, the mucosal lining hinders the mucosal permeation, retention, and permanence of formulations.^9^ To address these issues, in the present work, prepared bi-formulations, *i.e.*, MTF-loaded mucus-penetrating particles (MTF-MPPs) and MI-loaded mucus-penetrating particles (MI-MPPs), which were incorporated in a single system as a gel. MPPs-gel is expected to act as an improved therapeutic intervention for both MTF and MI intravaginal drug delivery.

The prepared MPPs are surface-modified, neutral surface-charged nanoparticles, which are expected to permeate the thick mucosal lining, thus contributing to enhanced vaginal retention and permanence.^10^ The neutral surface charge of the particles can reduce the cervicovaginal mucus bio-adhesion and steric hindrance to overcome the natural barrier and reach the targeted ovarian tissues. MPPs were developed using a biodegradable and biocompatible polymer, PLGA (poly lactic-*co*-glycolic acid), and a non-ionic surfactant, Pluronic F127 (PF127), which mainly neutralized the surface charge of MPPs to mimic the natural environment of the cervicovaginal mucus to facilitate the intravaginal penetration and delivery of the drug to the ovaries.^11^ A double-emulsion method (w/o/w) was employed with few modifications for the preparation of MPPs. Their further incorporation in a gel was optimized using an integrated quality by design approach. The development of the formulation was initiated by identifying the quality target product profile (QTPPs), including the initial risk assessment (RA) of MPPs and FMEA (failure mode effect analysis) of the factors influencing the material attributes, process parameters and affected quality attributes. The RA study was followed by the study of selected critical quality attributes (CQAs), critical material attributes (CMAs), and critical process parameters (CPPs).^12^ The approaches followed were a structured optimization process involving statistical, analytical and risk-management methodology to ensure the design, development, and quality of this new pharmaceutical product.^13^

Hence, the this research presented a proof-of-concept for the development of a novel drug combination of MTF and MI in an MPPs-loaded intravaginal gel for the amelioration of PCOS.

## 2. Material and methods

### 2.1 Materials

MTF was procured from Sun Pharma, Gurugram, Haryana, India as a gift sample. MI and Pluronic F127 were purchased from Sigma Aldrich Bangalore, India. Resomer RG 502 (PLGA 50 : 50) was procured from Evonik as a gift sample. Carbomer 974P was purchased from Lubrizol Advanced Materials, Inc. All other chemicals/solvents and reagents used in the experimental work were of analytical grade.

### 2.2 Animal ethical approval

The animal experimental protocol was approved by the Institutional Animal Ethics Committee (IAEC) of Jamia Hamdard; Registration No. 173/GO/ReBi/S/2000/CPCSEA (Protocol No. 1728). Female Wistar rats of 150–200 g (10–12 weeks old) were used in the experimental studies according to the PCOS model requirement. All the animals were kept under isolated environmental conditions, *i.e.*, temperature of 23 °C ± 2 °C, relative humidity of 60% ± 15%, and 12 h light/dark cycle with proper standard rodent diet and RO water ad-libitum. All procedures were carried out under the ARRIVE guidelines (Animal Research: Reporting of *In Vivo* Experiments).

### 2.3 Docking studies

Molecular *in silico* docking studies were conducted on therapeutically active drug molecules to determine the interaction with the targeted insulin and phosphor-inositol tris phosphate epimerase catalytic ligand-binding site of the receptors using the Schrodinger Maestro, version 9.6 software. The *in silico* study was used to assess the binding mode of the drug compounds on the targeted area, *i.e.*, the ovaries. To better understand the binding mode of MTF and MI (ligand molecules) at the molecular level, we carried out molecular docking simulations of the two compounds at the insulin and inositol receptor catalytic ligand binding sites (PDB ID: 1ir3 and 3uj4), respectively.^14^ The 3-dimensional (3D) ligands were stretched and transferred to the mol-sd file, and then entered into the LigPrep module for the docking study at the molecular level through the LigPrep method. Apo-proteins and the receptor grid were taken from the RCSB protein data bank, which was developed by removing the solvent by inserting a hydrogen molecule and diminishing the energy using the protein development wizard panel. The ligand was bound with the active site of the protein, which was assessed using the site map, and the receptor grid was further generated over the selected amino acid residue technique through the receptor grid forming panel (Glide version 6.9). Glide-ligand docking was conducted using the interaction of the developed ligand molecule and receptor glide, as analyzed by the docking score using the Glide extra-precision (XP) mode, with up to three poses saved per molecule (OPLS-2005 force field).^15^

### 2.4 Preparation of mucus-penetrating particles (MPPs)

**Fig. 1:**
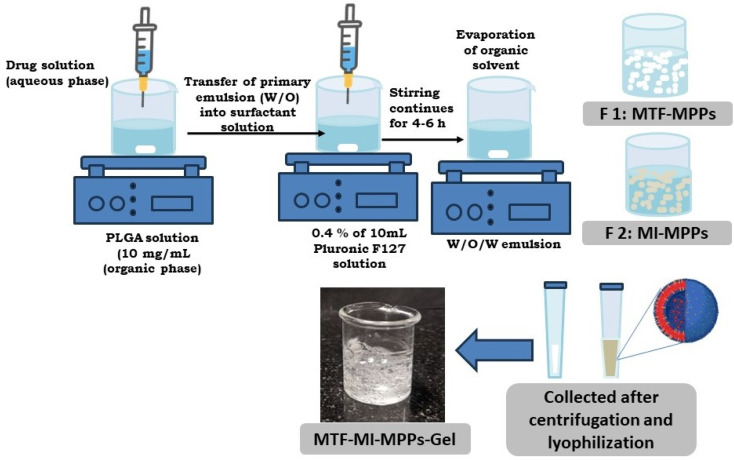
A schematic illustration of the methodology for the preparation of the bi-formulation MPPs incorporated in a gel. The double-emulsion method was applied in the fabrication of the MPPs.

The surface-modified PLGA nanoparticles (MPPs) were fabricated using a previously reported double-emulsion method with a few modifications, as described in Fig. 1. PLGA (30 mg) was solubilized in 3 mL acetone (10 mg mL^−1^), constituting the organic polymeric phase. The primary w/o emulsion was prepared by injecting 0.3 mL of drug aqueous solution (equivalent amount of 50 mg MTF pere MI drug) in the organic polymeric phase with a stirring speed of 1000 rpm at 80 °C.^16,17^ Subsequently, the primary phase of the w/o emulsion was added dropwise to 10 mL of aqueous surfactant solution (0.4% w/v of Pluronic F127) at a flow rate of 1 mL min^−1^. The temperature was maintained at 60–70 °C, while the stirring speed was set at 1500 rpm to make a w/o/w emulsion. The stirring was continued for 4–6 h to completely remove the organic solvent to yield MPPs. Then, the prepared MPPs were centrifuged at 10 000 rpm for 20 min and washed with Mili-Q water. Finally, they were lyophilized and stored for further studies. Furthermore, the lyophilized MPPs were incorporated in a gel, as described below, to prepare the MPPs-gel formulation.^18^

### 2.5 Development of formulation using the QbD approach

In the initial risk assessment, the QTPPs were decided and the effect of material attributes (MAs) such as polymer concentration, surfactant concentration, solvent volume, drug concentration, and other excipients were studied.^19^ Additionally, the process parameters (PPs) such as the effect of stirring speed, stirring time, temperature, solubility, injection type, and injection rate were assessed. These factors had a significant impact on the quality attributes (QAs) such as particle size, PDI, zeta potential, drug entrapment efficiency, drug loading capacity, drug release, and rheology. The above-mentioned factors that seem to influence the QAs were classified and assembled in an Ishikawa diagram (Fishbone diagram) as the initial risk assessment factors.^20^ These factors were further screened for final risk assessment and identified based on a severity score. Further, the risk priority number (RPN) was calculated for the given factors equivalent to the product of severity, probability of occurrence, and probability of detectability that facilitated the importance of risk factors such as MAs/PPs, which significantly affect the QAs.^12,21^

The final assessment of the risk factors (MAs and PPs) was comprised of two steps, firstly the relationship among the QAs concerning MAs/PPs was ranked based on levels such as high, medium, and low, and then a risk assessment matrix (RAM) was created for QAs-MAs/PPs. The results of the final risk assessment were interpreted using a Pareto chart, in which the high- and medium-risk QAs were designated as CQAs. In context of CQAs, different trials of placebo formulation were designed and characterized to optimize the PPs and screen an acceptable range of CMAs.^20,22^

#### 2.5.1 Formulation optimization by central composite design (CCD)

Based on the above-mentioned outcomes, the bi-formulation (MTF-MPPs and MI-MPPs) was further optimized, where CCD was implemented using the Design of Expert software (Stat-Ease, Minneapolis, USA). For the validation of the CCD model regarding our predicted goals, the bi-formulation was further post-analyzed in triplicate for the development of an accurate and precisely optimized formulation.^23^

### 2.6 Characterization of optimized MPPs formulation

#### 2.6.1 Particle size analysis, zeta potential, morphology of the particles, and % entrapment efficiency (EE)

The mean particle size, PDI, and zeta potential (surface charge) of MPPs were assessed using a Malvern Nano Zetasizer (Malvern Instrument, Inc. UK). An MPPs suspension was diluted 10 times using Milli Q water and further analyzed. The experiment was performed in triplicate. The morphology of MPPs was observed using transmission electron microscopy (TEM) on a TEM instrument (Tecnai G2 S-Twin, The Netherlands) at a high-resolution scale of 200 nm and precision power of 120 kV.^24^ Briefly, a small drop of the diluted sample was placed on a copper grid and dried at room temperature, which was placed on the grid port of the TEM. The size and shape of the particles of the sample were analyzed and captured using the Olympus Viewer imaging software. These factors were used to determine the suitability, homogeneity, physical stability, and surface charge of the formulation by assessing the mean particle size, PDI, and zeta potential.^25^

To determine the % EE, 10 mL of the prepared formulation was centrifuged at 10 000 rpm for 20 min at 4 °C. The free drug present in the supernatant was analyzed using LCMS and the % EE was determined using the following formula:
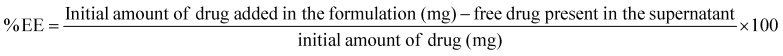


#### 2.6.2 Differential scanning calorimetry (DSC) analysis

To investigate the thermal instabilities and melting point of MFT-MI, drug-excipient and admixture of MTF-MPPs & MI-MPPs were subjected to DSC (Pyris 6, PerkinElmer Inc., USA). The MPPs were lyophilized in a Labconco Lyophilizer instrument at a temperature of −50 °C and pressure of −0.01 MPa for 72 h. Further, the lyophilized sample and other samples were exposed to a temperature in the range of 30–400 °C under a constant flow of nitrogen gas and a heat flow rate of 5 °C min^−1^.^26^

### 2.7 Preparation of MPPs-gel and conventional gel

Pre-sieved Carbomer 974P (by mesh number 100) was solubilized in the required volume (1% w/v) of Mili Q water at 40 °C for up to 6 h. Then, it was further sonicated for 10 min to remove air bubbles and 2–3 drops of triethanolamine (TEA) added to the solution to neutralize the prepared gel. For the optimum vaginal environmental conditions, 0.2 mg mL^−1^ w/v glycerol and the required amount of lactic acid were added with slow stirring to obtain a clear gel. The pH was adjusted between 3.5–4.5 using glycerol and lactic acid to regulate the acidic environment, which keeps the vagina non-irritating and moist.^27^ Subsequently, the lyophilized MTF-MPPs and MI-MPPs were incorporated in the gel with proper mixing for the uniform distribution of the particles. A conventional gel was also prepared using a similar method, in which the drug was loaded instead of nanoparticles.

#### 2.7.1 Texture analysis

Texture analysis is one of the important parameters for the estimation of a gel, where its force *versus* time graph represents the degree of firmness, density, cohesiveness, and consistency of the gel. The texture properties of the gel were estimated using a texture analyzer (TA) (XT-Plus, Stable Micro System Ltd, Surrey, UK). The probe disc having a diameter of 40 mm was moved down into the gel sample at a constant speed of 5 mm s^−1^ at a distance of 10 mm, and then moved upward at the same speed. This study was repeated thrice and a force *versus* time graph plot was generated. The data analysis was performed using the Texture Expert software.^28^

#### 2.7.2 Rheological studies

Rheological studies were performed to reveal the viscoelastic behaviour of the MPPs-incorporated gel (MPPs-gel) using an MCR 101 dynamic rheometer, Anton Paar (Germany) fitted with a cone and plate (CP-50-1) and their geometry (PP: 2–5) determined using the Rheoplus software. All studies were conducted at 25 °C. The flow behaviour of the MPPs-gel was assessed by the flow curve test and the apparent viscosity of the sample was observed using the Rheoplus software. This study was conducted to analyze the viscosity (Pa s) and shear stress (*τ*) as a function of shear rate (s^−1^) in the range of 0.1 to 100 s^−1^. An amplitude sweep test was performed to estimate the viscoelastic behaviour of the sample, where the linear viscoelastic region was estimated by calculating the storage modulus (*G*′) and loss modulus (*G*′′) based on the change in shear stress in the range of 0.05–100 Pa at a constant frequency of 1 Hz. Additionally, an oscillatory frequency sweep test was performed, where the storage modulus (*G*′), loss modulus (*G*′′), and complex viscosity (*η*) were estimated at the constant strain of 1% and change in frequency from 100 to 0.01 Hz.^29^

### 2.8 *In vitro* release studies

The *in vitro* release was estimated for 120 h using a dialysis membrane. Briefly, the sample was filled in an activated dialysis bag having a pore size of 25 Å with a molecular weight cutoff of 12 000 Da, which was further sealed on both sides. The optimized formulation (MPPs-gel) was compared with the conventional gel and MPPs suspension. The sample-loaded bag was immersed in 50 mL of simulated vaginal fluid (SVF) medium.^30^ Furthermore, the samples were placed in an incubator shaker at 37 °C and 100 rpm. At pre-determined intervals, 1 mL aliquots were taken out from the dissolution medium and immediately replaced with fresh medium to maintain the sink condition. The collected samples were analyzed by the developed LCMS analytical method.^31,32^ The details of the different samples are as follows:

Sample A: MI-MTF-gel (conventional gel),

Sample B: MTF-MI bi-formulation MPPs suspension (admixture of MFT-MPPs and MI-MPPs) and

Sample C: MTF-MI bi-formulation MPPs-gel (admixture of both formulations incorporated in gel).

### 2.9 *Ex vivo* studies using vaginal tissue of goat

The freshly excised mucosal vaginal membrane of a goat was collected from the slaughterhouse and cleaned with saline solution.^33^ The goat vaginal mucosa tissue sample was cut with a size of 1.24 cm^2^. This was used to estimate the vaginal permeation study of the following samples: conventional gel, bi-formulation MPPs suspension, and MPPs-gel. Briefly, the vaginal tissue was placed on the open mouth of a Franz diffusion cell assembly having an effective diffusion area of 0.785 cm^2^.^34^ The open mouth of the Franz diffusion cell assembly had an effective diffusion area of 0.785 cm^2^. The receptor compartment was filled with 10 mL of SVF (pH 4.2), while the donor compartment was filled with the test sample. All the sample formulations were adjusted with an MTF and MI concentration of 15 mg.^35,36^ The corresponding assemblies were placed on a magnetic stirrer at 200 rpm and 37 °C temperature. Then, a 1 mL aliquot sample from each assembly was withdrawn and replaced with fresh SVF at a predetermined interval for 8 h. The samples were analysed by LCMS. The vaginal permeation flux of the respective drug was estimated through its *ex vivo* permeation release profile. The intravaginal delivery of the drugs was studied using the required permeation parameters such as steady state flux, *J*_ss_, and permeability coefficient, *K*_p_. The slope of the linear plot of the permeation represents the *J*_ss_ value of the respective drug in the different formulation samples.
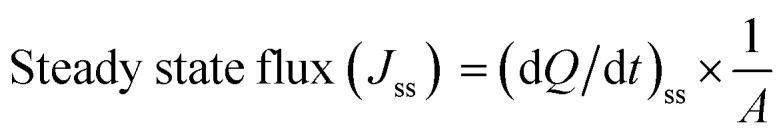
where d*Q*/d*t* represents the rate of change of cumulative intravaginal drug content (*Q*) as a function of time (*t*) and *A* represents the effective diffusion area. The linear regression fit value of the curve was obtained from the slope, d*Q*/d*t*.Permeability coefficient (*K*_p_) = *J*_ss_/*C*_d_where *C*_d_ is the drug concentration, which remained constant.

### 2.10 Confocal microscopic study

The penetration ability of the optimized MPPs and MPPs-gel through the vaginal mucosal membrane was determined and compared with the conventional gel. They were mounted on the mouth of a Franz diffusion cell (FDC) filled with simulated vaginal fluid media, SVF (pH 4.2). Furthermore, 1 mL of rhodamine B-loaded conventional gel was filled in the donor compartment of the FDC, while 1 mL of rhodamine B-loaded test samples was poured in the donor compartment of another FDC. These cells were kept on a magnetic stirrer at a temperature of 37 °C and stirring speed of 200 rpm. This experiment was continued for approximately 6 h, which was expected to be sufficient time for the diffusion of the dye across the tissue.^14^

### 2.11 Irritation studies on vaginal tissue

For the determination of irritation potential on the vaginal tissue of female Wistar rats with an average weight of 150–200 g, the different groups (*n* = 3) were treated and compared with the control group as mentioned below. After one week of treatment, the animals in each group were sacrificed and their vaginal tissue excised and immersed in 10% formalin solution, which was prepared in phosphate buffer or saline solution (pH 7.4).^37^ The vaginal tissue was treated with concentrated ethanol for dehydration, followed by dipping in xylene to prepare paraffin blocks. Further, a thin section of 5 μm was cut by microtome, and then processed for hematoxylin and eosin staining. The prepared slides were visualized and analyzed using an inverted microscope (Olympus IX71 inverted microscope at 10× and 40×, Tokyo, Japan).^38^ The effect of the test samples was demonstrated, interpreted, and compared with the control group.

Group A: negative control group kept as an untreated group.

Group B: test group of optimized MPPs suspension treated with 100 μL of the equivalent dose administered through the vaginal route with the help of an applicator (mixed suspension of MI-MPPs and MTF-MPPs of 1 : 1 ratio).

Group C: test group of optimized MPPs-gel treated with 500 μL of the equivalent dose administered through the vaginal route with the help of an applicator.

### 2.12 *In vivo* animal studies

#### 2.12.1 PCOS model development

Female Albino Wistar rats with an average weight of 150 g were selected for *in vivo* testing. The Wistar rats were randomly separated into four groups (*n* = 6), where Group A represented the negative control (healthy animals without disease induction and treatment). Group B represented the positive control (rats were exposed to a PCOS-inducing agent). Group C represented the PCOS-induced animals treated with intravaginal delivery of 0.5 mL conventional vaginal gel with a dose of 30 mg kg^−1^ MTF and 37.5 mg kg^−1^ MI (once a day). Group D represented the PCOS-induced animal group treated with intravaginal delivery of 0.5 mL MPPs-gel formulation with a dose of 30 mg kg^−1^ MTF and 37.5 mg kg^−1^ MI (once a day).

Mifepristone solution in sesame oil was administered orally with a dose of 20 mg kg^−1^ once a day for 21 consecutive days. All animals were monitored for their estrous cycle on the last week of mifepristone administration and a continuous dioestrus phase was considered for PCOS induction.

#### 2.12.2 Treatment pattern and *in vivo* analysis

The animals were monitored for the presence of a continuous destroys phase in their estrous cycle and when observed in the rats, the animals assigned to different groups as envisaged in the study plan were subjected to the treatment framework for 28 consecutive days. For the estimation of the pre- and post-treatment effect on the estrous cycle, vaginal smears were taken for 2 weeks pre- and post-treatment at a fixed time daily. The phases of the estrous cycle were analyzed by microscopy, which focused on the different types of cells present in the sample slides of vaginal smears following methylene blue staining. Proestrus was identified by round, nucleated epithelial cells; estrous illustrated by cornified squamous epithelial cells; metestrus was identified by epithelial cells and leukocytes; and dioestrus was validated by the presence of nucleated epithelial cells and a predominance of leukocytes. On the 30th day of treatment, the animals were sacrificed and their biochemical parameters were estimated. The animal blood samples of the corresponding group were immediately centrifuged at 5000 rpm for 10 min at 4 °C for the serum estimation. The levels of testosterone, estradiol, luteinizing hormone, and insulin were determined by enzyme-linked immunosorbent assay kits. Subsequently, the animal ovaries were excised and their average weight was determined.^39^

## 3. Results and discussion

### 3.1 Molecular docking

**Fig. 2:**
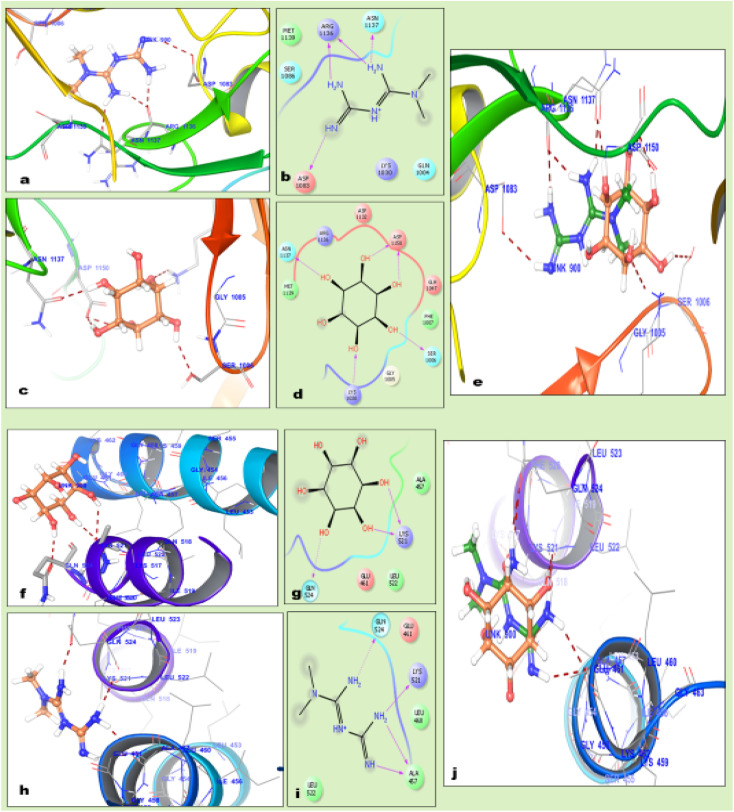
2D representation depicting the hydrogen bond interaction of the MFT (metformin) and MI (myoinositol) drug molecules with protein: (a) binding pattern of MTF against insulin receptor, (b) 2D structure of MT, (c) bonding interaction of MI with the insulin receptor, (d) 2D structure of MI, (e) superimposition of MI with MTF against the insulin receptor protein, (f) binding pattern of MTF against the inositol receptor, (g) 2D structure of MI, (h) bonding interaction of MTF with the phospho-inositol tris kinase (PI3K) protein (inositol receptor), and (j) superimposition of MI with MTF against inositol receptor protein.

Molecular docking is advanced computational *in silico* technology for the selection of precise drug delivery systems to ensure the hypothesized concept, which was applied in the design of the formulation. The docking results revealed that MTF is bound tightly in the active site of the insulin receptor protein and forms hydrogen bonds with Arg1136, Asn1137, and Asp1083, while inositol forms hydrogen bonds with Asn1137, Asp1150, Lys1030 and Ser1006, as mentioned in Table 1. These interactions were also validated by the superimposition of inositol with metformin, which gave the same interaction in a catalytic domain, as shown in Fig. 2.

**Table 1:** Molecular docking scores and bonding interaction of protein

Drug	Protein	Docking score	Interactions	
			H-Bonding length	Other bonds
MTF	Insulin receptor	−8.142	Arg1136, Asn1137 and Asp1083	SER 1006
MI	Insulin receptor	−5.674	Asn1137, Asp1150, Lys1030 and Ser1006	GLY 1005
MI	Inositol receptor	−6.881	Lys521 and Gln524	GLU461, ALA457, Ser 455, Jle 456, Leu 453, Unk 900
MTF	Inositol receptor	−4.464	Ala457, Lys521 and Gln524	LEU523, ILE519, GLN518, GLU461, LEU460, ILE456, GLY458, LEU 453

The docking results also revealed that MI is tightly bound in the active site of the inositol receptor protein and forms hydrogen bonds with Lys521 and Gln524, while MTF forms hydrogen bonds with Ala457, Lys521 and Gln524. These interactions were also validated by the superimposition of MTF with MI, which showed the same interaction in the catalytic domain and inositol having a higher docking score than metformin, as described in Table 1.

Combination therapy of MTF and MI has been appreciably effective in the treatment of PCOS, as supported by significant research. However, this combination has not been explored as a nano-appended intravaginal drug delivery system to date. The reports in the literature prove the availability of binding receptors on the targeted ovaries. Furthermore, the hypothesized research on the development of mucus-penetrating particles showed potential to be successful based on the confocal microscopic study and *ex vivo* permeation study.

### 3.2 Optimization and development of bi-MPPs gel using QbD approach

**Fig. 3:**
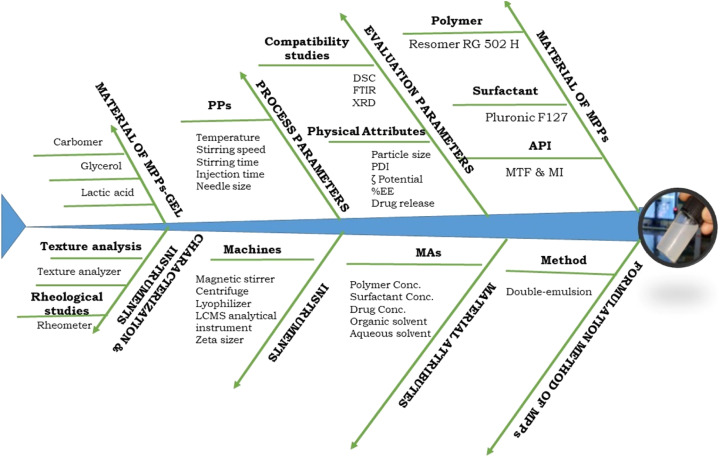
The Ishikawa or fishbone diagram demonstrating the potential factors essential for the design and development of MPPs-gel.

The quality by design (QbD) process is associated with patient-centric QTPPs (quality target product profiles), which focuses on the quality of the product, human safety, and therapeutic efficacy of the pharmaceutical dosage form. In the present study, the QbD approach was applied in three phases, *i.e.*, risk assessment (initial and final), design of expert for optimization of high-risk factors, and model validation used to validate the predicted and experimental data. A fishbone diagram was designed to classify the required factors, including therapeutic factors, materials characteristics, formulation processing factors, and method validation, as shown in Fig. 3. All these together reflect the quality of the pharmaceutical product.

The initial risk assessment revealed the QTPP elements and their potential impact on the quality of the pharmaceutical formulation, where the quality targets and justification are discussed for each QTPP element according to the literature survey, experimental findings, and previous laboratory trials, as shown in Table S1.† The critical quality attributes (CQAs) were also observed to confirm the quality standard of the formulation. Alternatively, the QTPPs also depicted the required quality standards based on failure modes and the related reasons through the FMEA (failure mode effect analysis),^40^ where different risk factors are involved in the development of the formulation. The severity score (*S*), probability of occurrence (*O*) and detectability (*D*) scores of MAs and PPs regarding their failure mode were revealed. This scoring system, as mentioned in Table S2,† was provided with segregated data depicting the potential risk that had an effect on the formulation development. The risk priority number (RPN) denotes the total score used to identify the potential risk-affected formulation development. The score obtained from RPN was classified based on risk, including low risk of QAs (0–150), medium risk of QAs (150–300), and high risk (300–450). Further, the fixed range of RPN associated with high, medium, and low risk was identified accordingly, which seemed to have significant critical effects on the desired quality, therapeutic effect, administrable route, and safety aspects, as mentioned in ESI, Table S2.† These associated RPN scores of high-risk factors were also designated as critical quality attributes (CQAs). Moreover, the final risk assessments were estimated by RAMs.

A final RAM (risk of material attribute) was developed for the relationship between CQAs and MAs/PPs after the risk reduction for the final risk assessment, as mentioned in Fig. S1 and S2.† The RAM matrix facilitated the QAs *vs.* QTPPs relationship. The generated risk assessment matrix (RAM) of QA-MAs/PPs signified the inter-dependence effect based on the rating.

**Fig. 4:**
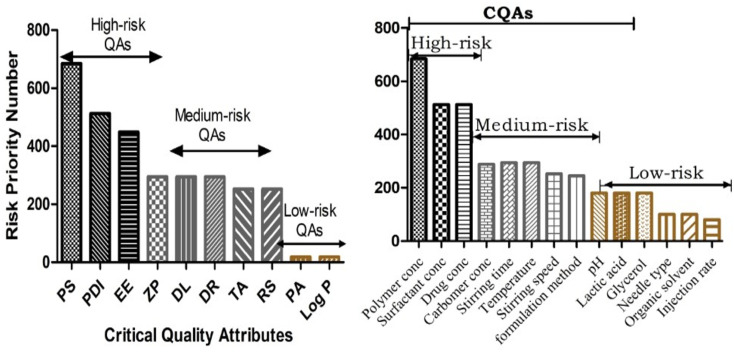
Pareto chart of CQAs and MAs/PPs for RPN.

Based on the screening of the MAs about the required QAs, a new RAM of the selected MAs was developed in the form of a Pareto chart of QAs-MAs/PPs (Fig. 4). The PLGA concentration, PF127 concentration, and drug concentration were high-risk MAs (CMAs), while the org/aq. phase ratio, temperature, stirring speed, and stirring time formulation method were medium risks of PPs. Also, the organic solvent, needle type, and injection rate were found to be low-risk MAs/PPs. We analysed that in the method for the development of the formulation, three physical attributes, *i.e.*, PS, ZP, and EE, were at higher risk in CQAs together with high severity scores. In the CQAs, high and medium risks of MAs/PPs were represented as CMAs/CPPs and their resultant responses were considered in the experimental studies, as revealed in Fig. 4.

All the final risk assessments were analysed and interpreted, where the various placebo formulation trials were performed by changing one attribute, *i.e.*, either material or process parameter, by different concentrations or modes, as mentioned in Table S3† in the bold letter, while the other attributes were remained in constant mode and values. Further, the effect of all the given factors was examined, as mentioned in Table S2 (ESI†). This helped to screen the precise range of concentration of all the materials. Moreover, based on their responses, all the process parameters were optimized, as mentioned in Table S3.†

### 3.3 Formulation optimization by CCD

Given that the CQAs were determined and interpreted in the trial formulations, three important CMAs (factors) were used as independent variables for, MPPs, *i.e.*, MFT-MPPs (D1) and MI-MPPs (D2). They were analysed by the six CQAs (responses), in which three responses were designated for MTF-MPPs (D1) and the other responses were designated for MI-MPPs (D2). The other factors were kept constant as some of the process parameters (PPs) were optimized during the screening of the trial formulations. Three CMAs such as PLGA polymer (X1), surfactant (X2), and drug (X3) were taken as independent factors, where five levels were generated in the CCD, as mentioned in ESI, Table S4.†^23^

Alternatively, six responses of dependent variables were included in the CCD process including particle size of D1(Y1), particle size of D2 (Y2), PDI of D1 (Y3), PDI of D2 (Y4), EE (encapsulation efficiency) of D1 (Y5) and EE of D2 (Y6). A total of 20 runs was generated, whose responses were determined using ANOVA, as revealed in Table 2. Surface plots of all the responses (CQAs) were created and the effect of these variables on the given responses of both metformin (D1) and myoinositol (D2) was interpreted. The other CPPs were taken as constant with fixed values given that these CQAs met the required targets in the trial formulations. The goal and limits of responses were associated with the targets of CQAs used to select the optimized formulation. All the responses were analysed to fit the respective polynomial models.

**Table 2:** Details of all the independent variables generated in the CCD and their effect (responses) on the CQAs of the optimized formulation

Std	Run	Factor 1	Factor 2	Factor 3	Response 1	Response 2	Response 3	Response 4	Response 5	Response 6
		A: PLGA Polymer (mg)	B: PF127 (%)	C: MTF and MI (mg) same quantity for individual drugs	Particle size of MTF (D1) (nm)	Particle size of MI (D2) (nm)	PDI of MTF (D1)	PDI of MI (D2)	% EE of MTF (D1)	% EE of MI (D2)
7	1	20	0.6	60	187.2	188.2	0.116	0.099	50.46	52.12
6	2	40	0.2	60	254.4	256.12	0.389	0.381	67.14	65.85
8	3	40	0.6	60	247.7	248.2	0.234	0.201	64.45	65.12
4	4	40	0.6	40	245.6	247.6	0.136	0.231	68.29	69.01
9	5	13.18	0.4	50	175.8	176.4	0.182	0.208	44.89	49.5
10	6	46.82	0.4	50	289.6	288.5	0.355	0.343	68.57	69.97
14	7	30	0.4	66.82	196.8	197.4	0.159	0.15	57.85	58.49
13	8	30	0.4	33.18	195.8	196.9	0.173	0.201	65.14	66.13
11	9	30	0.04	50	235	221.7	0.346	0.356	65.23	65.21
1	10	20	0.2	40	185.5	184.9	0.261	0.261	61.61	58.26
12	11	30	0.74	50	200.9	188.9	0.146	0.164	61.95	59.87
18	12	30	0.4	50	195	198.5	0.17	0.153	66.25	67.13
19	13	30	0.2	50	205	206.8	0.26	0.248	66.98	66.25
5	14	20	0.2	60	192.6	194.2	0.145	0.231	53.98	56.25
3	15	20	0.6	40	181.9	180.8	0.201	0.241	56.74	56.03
2	16	40	0.2	40	254.2	252.8	0.34	0.312	69.84	68.91
15	17	30	0.6	50	194.9	196.5	0.141	0.145	63.85	63.74
20	18	30	0.4	60	210.5	220	0.168	0.164	62.22	64.11
16	19	30	0.4	40	195.8	197.2	0.175	0.175	66.18	66.92
17	20	30	0.6	60	202.8	203.4	0.14	0.094	60.48	60.23

Based on the estimated data of all the CQA responses of the related runs, as mentioned in Table 2, polynomial models of best-fitted statistics were examined concerning the sequential *p*-value, which was expected to be of the highest significance, while the *R*^2^ value, adjusted *R*^2^ value, and predicted *R*^2^ were also expected to be high but their difference must be less than 0.2 and the adequate precision expected to be greater than 4. The contour plot of all the responses depicted the best-fitted polynomial model, as mentioned in Fig. S4 (ESI†). The framework of these plots indicated the effects of PLGA concentration, PF127 concentration, and drug concentration on the critical quality attributes of the different responses.^41^

Different polynomial mathematical equations were developed using the appropriate software and the CPPs were correlated with CMAs. The high and low levels of the given factors were coded by +1 and −1, respectively, which were used to determine the relative impact of the independent variables compared to their coefficients.^42^

#### 3.3.1 Effect of the independent variables on the size of the particles

**Fig. 5:**
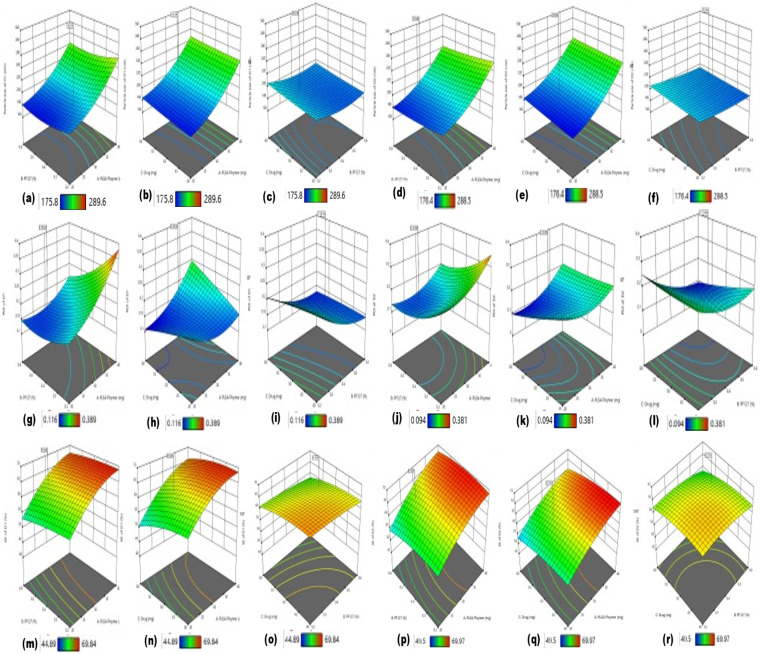
3-D surface plots of the optimization of the formulation representing the effect of independent variables. Here, graphs (a), (c), (e), (g), (i), (k), (m), (o) and (q) represent the 3-D surface plots of MTF-MPPs, while graphs (b), (d), (f), (h), (j), (l), (n), (p) and (r) represent the 3-D surface plots of the MI-MPPs formulation.

Particle size (PS) is considered to be a critical parameter in the CQAs in the fabrication of MPPs, given that it facilitates the penetration and release of the therapeutic drugs. The quadratic equations of particle size for metformin (D1) and myoinositol (D2) were generated in relation to the effect of material attributes (MAs). A graph was plotted for the predicted value *versus* the actual value of both drugs individually (D1 and D2), as mentioned in Fig. 5.PS(D1) = 196.77 + 32.66 × *A* − 5.78 × *B* + 2.07 × *C* − 0.7875 × *AB* − 1.26 × *AC* + 0.1758 × *BC* + 13.01 × *A*^2^ + 7.35 × *B*^2^ + 0.9024 × *C*^2^PS(D2) = 202.19 + 32.60 × *A* − 5.55 × *B* + 2.94 × *C* − 0.3775 × *AB* − 1.60 × *AC* − 0.3617 × *BC* + 11.95 × *A*^2^ + 2.12 × *B*^2^ + 0.3348 × *C*^2^

In these equations, the independent factor terms are concentration of PLGA polymer (*A*), PF127 (*B*), and drug (*C*), whereas *AB* defines the PLGA polymer and PF127.

All the variables and interaction terms that indicated negative values in both equations facilitated negative effects in terms of concentration on the particle size, whereas all the variables and interaction terms that exhibited positive values depicted positive effects on the particle size, indicating that as the concentration of both PLGA and PF127 increases, the particle size of the developed formulation increases. However, in both the equations, variable *A* depicts a high positive value of the coefficient variables, indicating that the PLGA polymer has a greater effect on the particle size than variable *C*, which has less effect on particle size. Alternatively, variable *B* and interaction *AC* showed negative values in both equations, which exhibited a negative effect on particle size, as mentioned in Fig. 5. Henceforth, the developed average particle size range for D1 was 175.8 to 289.6 nm, while that of D2 was in the range of 176.4 to 288.5 nm, as observed in Table 2.^43^

#### 3.3.2 Effect of the independent variable on polydispersity index (PDI)

The effect of all three critical material attributes on PDI was determined for the assessment of the CQAs. The given polynomial quadratic equation of D1/D2 was generated for the PDI of MPPs.PDI(D1) = 0.1735 + 0.0488 × *A* − 0.0578 × *B* − 0.0055 × *C* − 0.0338 × *AB* + 0.0435 × *AC* + 0.0097 × *BC* + 0.0331 × *A*^2^ + 0.0252 × *B*^2^ − 0.0031 × *C*^2^PDI(D2) = 0.1581 + 0.0381 × *A* − 0.0542 × *B* − 0.0153 × *C* − 0.0136 × *AB* + 0.0264 × *AC* − 0.0276 × *BC* + 0.0422 × *A*^2^ + 0.0363 × *B*^2^ + 0.0068 × *C*^2^

The negative coefficient variable exhibited an inverse effect on the PDI of the particles. Variable *B* possessed a higher value than *C*, which means variable *B* imparts a more remarkable effect on the PDI in both equations. Similarly, coefficient variable *A* showed a positive value, which indicates that variable *A* is directly proportional to the PDI but the interaction of *AB* and *BC* possesses a negative effect, as seen in Fig. 5. According to previously reported data, the PDI of ∼0.3 is predicted to be the optimum value for nanoparticles. The PDI of all the runs of both drugs was observed to be ≤0.4, *i.e.*, the PDI of D1 was in the range of 0.116–0.389 and that of D2 in the range of 0.094–0.381, as shown in Table 2.^44,45^

#### 3.3.3 Effect of the independent variable on the % entrapment efficiency (EE)

The % EE was analysed by the quadratic equation developed in the CCD% EE(D1) = +65.80 + 6.35 × *A* − 1.35 × *B* − 2.33 × *C* + 0.5188 × *AB* + 0.9213 × *AC* + 0.0416 × *BC* − 2.95 × *A*^2^ − 0.5020 × *B*^2^ − 1.30 × *C*^2^% EE(D2) = + 66.85 + 5.91 × *A* − 1.19 × *B* − 1.83 × *C* + 0.7163 × *AB* − 0.1287 × *AC* − 0.3632 × *BC* − 2.46 × *A*^2^ − 1.52 × *B*^2^ − 1.54 × *C*^2^

In the given equations, the negative coefficient variables *B* and *C* had an inverse effect on the entrapment efficiency of D1 and D2, indicating that as the concentration of surfactant increases, the entrapment efficiency of the drugs decreases. The high value of variable *A* indicates its positive effect, which showed that as the concentration of polymer increases, the EE is also enhanced.^46^

The CQA responses were identified and studied based on the fit of the respective polynomial models, where the best-fitted responses were demonstrated concerning the highest significant *p*-value, high *R*^2^ values of adjusted data and predicted data, as mentioned in Table S6 (ESI†). The contour plots of the respective MPPs formulations in relation to the CQAs based on the best-fitted model of the different responses are shown in Fig. S3 (ESI†).

The particle size, PDI, and EE of D1 and D2 increased as the concentration of the PLGA polymer (*A*) increased from a lower limit to a higher limit. However, all these CQAs were reduced as the concentration of surfactant increased from a low limit to high limit. These effects are attributed to the anticipated reduction in interfacial tension in the organic phase and aqueous phase, which also eventually affects the % EE, as observed in Fig. 5.^47,48^

In accordance with the above-mentioned outcomes of all the runs, the optimum formulation of both drugs with the highest desirability expected by the CCD model was generated, where 30 mg of PLGA, 0.4% w/v of PF127 and 50 mg of the respective drug were used. To validate the CCD model and confirm the accuracy and precision of the predicted model, the post-analysis of the formulation was performed thrice with the predicted conditions. In the post-analysis of CQAs, the mean experimental values of all the given responses were compared with the predicted values, as mentioned in Table S4.†

**Fig. 6:**
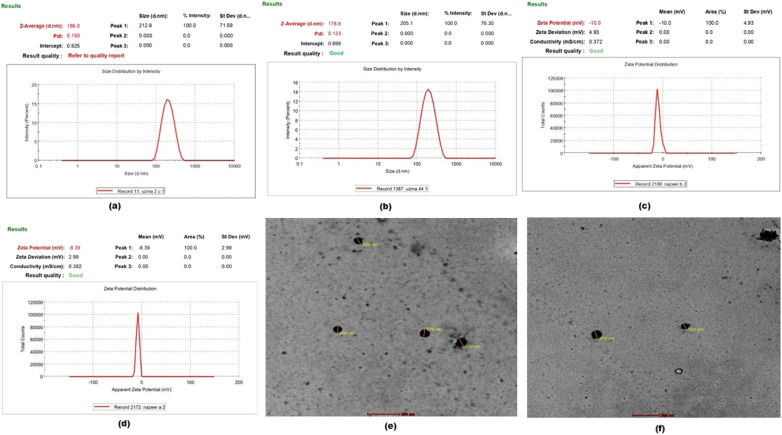
Characterization parameters of the optimized formulations: MTF-MPPs and MI-MPPs. (a and b) Particle size and PDI analysis graph; (c and d) zeta potential analysis data and (e and f) TEM-micrograph of MTF-MPPs and MI-MPPs formulations.

For the particle size analysis, the obtained outcome of the optimized MTF-MPPs formulation with given concentrations of drug and excipients was *Z*-average particle size of 195.0 nm in the desired range of less than 200 nm. The obtained PDI was 0.150, with a predicted desired range of 0.05 to 0.3. Also, the % EE was estimated to be 66.25% with a maximum predicted goal, as validated in Fig. 6(a). Further, the estimated particle size for the optimized MI-MPPs formulation was *Z*-average particle size of 178.8 nm with PDI of 0.123 and EE of 67.13% (Fig. 6(b)). The post-analysis could be extrapolated to conclude that the change in particle size, and PDI fell within the design space.^44^ The outcomes of the particle size estimation were authenticated by the graph presented in Fig. 6(a) and (b).

### 3.4 Characterization of optimized MPPs formulation

#### 3.4.1 Mean zeta potential, particle morphology and % entrapment efficiency (EE)

The nanoparticles were examined by measuring the zeta potential, which demonstrated the degree of repulsion between the particles based on their surface charge. According to a previous study, the zeta potential should be neutral or more than −10 mV, which is required for MPPs properties in the cervicovaginal mucus. For intravaginal drug delivery, the surface charge of particles plays a crucial role in their penetration through the barriers of the vagina, eventually leading to the delivery of the drug to the targeted area of the ovaries.^1^ The average *ζ* potential of the MTF-loaded MPPs and MI-loaded MPPs was found to be −5.19 mV and −6.45 mV, as seen in Fig. 6(c) and (d), respectively. Thus, the determined *ζ* potential of both formulations was within the acceptable limit.^49^ The obtained particle size and surface charge on the particles support the optimized formulations, ensuring that they would cross the mucosal layers and deeply penetrate them, eventually reaching the targeted area of the ovaries through the broad network of blood vessels *via* uterovaginal pathways. These optimized formulations were further characterized by TEM (transmission electron microscopy), and the corresponding TEM micrographs of MFT-MPPs and MI-MPPs were corroborated with the outcomes of the particle size analysis of the optimized formulations. This clearly showed that the MPPs have a uniform morphology with a spherical and smooth shape, as shown in Fig. 6(e) and (f), respectively. The majority of particles have a size of ∼200 nm, demonstrating their the suitable si†ze range for intravaginal drug delivery.^50^ The % EE of the optimized MTF-MPPs formulation was estimated to be 66.25%, as the maximum predicted goal. Similar outcomes were found for MI-MPPs with an average EE of 67.13%.^51^

#### 3.4.2 Differential scanning calorimetry (DSC) analysis

**Fig. 7:**
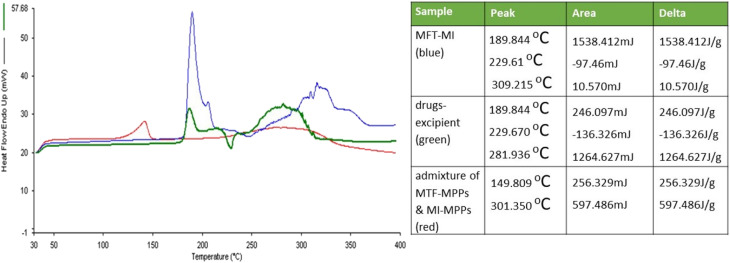
DSC graph differentiated by different colours, where the MTF-MI mixture is represented in blue, the drug-excipient mixture is depicted in green and bi-formulation is depicted in red.

An investigation was performed to study the compatibility between the dual drug admixture, drug-excipient, and bi-formulation in a 1 : 1 ratio. The graphs of both drugs, *i.e.*, MTF and MI, showed distinct peaks at 229.61 °C and 309.21 °C, respectively, as shown in Fig. 7. The data revealed that the peaks of both drugs were similar to their melting points, indicating that MTF and MI are compatible. Similarly, the drug excipients were found to be compatible given that there was no deviation in the peak values. Moreover, the bi-formulation showed a shift and slightly diminished drug peaks, which can be attributed to the process of encapsulation, yielding an amorphous nature in the drug. Also, the distinct drug peaks revealed that both drugs did not show any interaction.^52^

### 3.5 Preparation of MPPs-incorporated gel

The bi-formulation MPPs-gel was developed using 1% Carbomer 974P given that it ensures better cohesiveness, which is suitable for intravaginal delivery. The acidic pH of the vaginal environment is capable of causing structural changes by altering the viscosity of the carbomer gel. Thus, it is expected to provide a pathway to MPPs for the ease of penetration or deep permeation *via* the uterovaginal pathway. The developed gel was characterized by texture analysis and rheological studies, as discussed below.

#### 3.5.1 Tensile strength

**Fig. 8:**
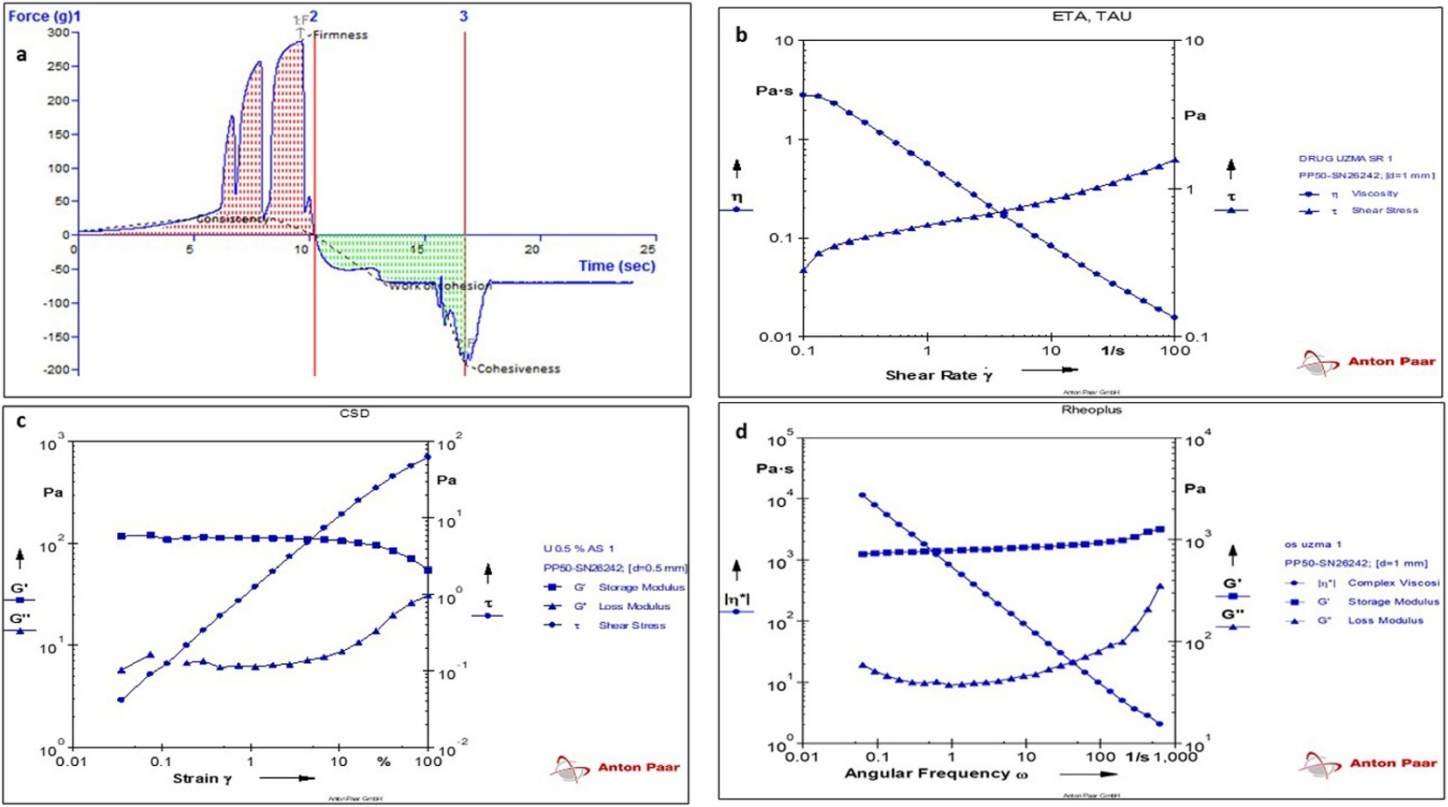
(a) Texture analysis of the MPPs-gel formulation. The rheological behaviour of the MPPs-gel formulation illustrated by different tests, such as (b) flow curve, (c) amplitude sweep test and (d) frequency sweep test.

The above-prepared formulation was further characterized by texture analysis for the assessment of its firmness, consistency, and cohesiveness. The texture curve, as seen in Fig. 8(a), in which the force was recorded to be 287 g, indicated the firmness of the sample, where the associated area (force × time) was found to be 831.20*g* s, indicating the consistency of the sample. Additionally, the force developed in the opposite direction was found to be −193.02*g*, which depicted the optimum cohesiveness and its area revealed the work of cohesion (−466.69*g* s), as mentioned in Fig. 8(a). The above-mentioned results from the enclosed texture curve indicate a uniform and smooth texture free from grittiness and lumps.^46,53^

#### 3.5.2 Rheological studies

Rheological studies were performed, where the flow property of MPPs-gel was determined using a rheogram. The shear stress and viscosity values were generated at different shear rates, which revealed the shear-thinning behavior of the MPPs-gel, as shown in Fig. 8(b). The rheogram from the amplitude sweep test, representing the storage modulus (*G*′) and loss modulus (*G*′′) on the *Y*-axis *versus* strain on the *X*-axis, appeared to be quite linear, as shown in Fig. 8(c). The result obtained from the amplitude LVR (linear viscoelastic region) test depicted the high storage modulus in relation to the loss modulus, further indicating that the high elasticity of MPPs-gel is maintained by virtue of the lower dissipation of energy. The obtained data from the frequency sweep study was used to analyze the inner changes in the structure/matrix of the gel. This was performed at 1% strain in the frequency sweep range of 1.0 to 100 Hz at room temperature, as shown in Fig. 8(d). The values of the storage modulus (*G*′), loss modulus (*G*′′), and complex viscosity (η*) were revealed according to change in frequency sweep. Thus, the logarithmic rheogram depicted that no crossover was observed and both *G*′ and *G*′′ increased, while the complex viscosity displayed linear reduction. The plot delineated the high efficiency due to the elevation of the modulus with respect to time. The MPPs-gel formulation was also determined in the temperature sweep up to 50 °C (ambient temperature), which did not show any abnormal behaviour. This indicates that the prepared formulation can be easily transported and stored at room temperature if it acquires a particular phase and viscosity.^43,44^

### 3.6 *In vitro* release studies

**Fig. 9:**
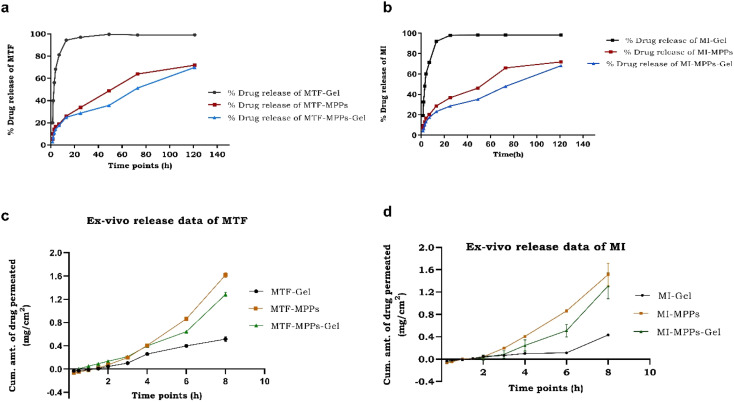
Comparative *in vitro* release permeation data of MTF (a) and MI (b). *Ex vivo* permeation of MTF (c) and MI (d) drugs through the vaginal tissue of an animal.

*In vitro* release is one of the crucial parameters to determine the release behaviour of drugs from the optimized formulation. As apparent from the QTPP parameters, the quality attributes successfully predicted the *in vitro* release status of a given formulation from the proposed intravaginal drug delivery system. The *in vitro* release of the MI-MTF-loaded conventional gel (sample A), MTF-MI bi-formulation MPPs suspension (sample B), and MTF-MI bi-formulation MPPs-gel (sample C) was performed at a predetermined time. It was observed that sample C released approximately 69.86 ± 4.65% of MTF and 67.14 ± 5.74% of MI from it within 120 h (5 days) compared with sample A, which was estimated to be approximately 94.89 ± 4.17% of MTF and 90.91 ± 15% of MI drugs released within 12 h, as shown in Fig. 9(a) and (b), respectively. Similarly, the *in vitro* release profile of sample B was estimated to be almost 71.87 ± 5.15% of MTF and 70.85 ± 3.97% of MI released within 120 h, which were observed to be sustained in a consistent pattern compared with MFT-MI-gel, as shown in Fig. 9(a) and (b), respectively. The conventional gel was used to validate the prolonged effect of the proposed optimized formulation because when the conventional gel comes in contact with the acidic pH environment of the vagina, it loses its structure, its viscosity decreases and the drugs can be released from the gel at a faster rate. Alternatively, the MPPs particles are composed of PLGA, a biodegradable polymer, which is used in pharmaceutical studies to maneuver a variety of release profiles for over weeks and months.^36,54^ The current study corroborates this and suggests the release of drugs for over 120 h/5 days. In contrast, MPPs-gel depicted a similar release pattern but its initial release was a bit delayed. This can be attributed to the presence of the sturdy carbopol gel matrix/structure, which kept the MPPs intact and did not allow them to interact with the vaginal milieu. However, as the interaction of MPPs-gel continues with the acidic pH environment, the gel loses its structure, its viscosity decreases and the particles are released from the gel mesh.^10^ This sluggish release in the initial phase was then followed by a sustained release. Owing to the proximity of the particles with the tissue interface, it is expected that the particles with a nanosize would easily penetrate the vaginal barriers and concentrate at the site of action, well above the minimum effective concentration. Thus, this occurred over a period of 5 days, eventually reducing the frequency of dosing, and also mitigating the drug toxicity.^49^

Moreover, the release kinetics of MTF and MI was determined and it was observed that the MPPs-gel formulation obeyed zero-order release kinetics, having an *R*^2^ value of 0.9 and 0.91, respectively. Also, the results showed that it followed the Higuchi matrix, having an *R*^2^ value of 0.96 and 0.98, respectively, which is also regarded as non-Fickian diffusion. Similarly, the release kinetics of the MTF and MI drugs from the MPPs formulation followed zero-order kinetics with the *R*^2^ value of 0.96 (MTF) and 0.94 (MI), and it also followed the Higuchi matrix, respectively. The reason for the above-mentioned release kinetics was the initial slow release for at least 3 h, and further a constant zero-order release pattern was observed, which is essential for the intravaginal drug delivery system of nanoparticles, given that it maintains the plasma concentration. Alternatively, the drug release kinetics of the MTF-MI-conventional vaginal gel was concentration-dependent and followed first-order kinetics, having an *R*^2^ value of 0.90 and 0.97, respectively. Thus, it followed the Korsmeyer–Peppas model, having an *R*^2^ value of 0.78 (MTF) and 0.85 (MI). The details of all the graph plots are mentioned in the ESI (Fig. S4 and S5†).

### 3.7 *Ex vivo* studies on vaginal tissue of goat

The purpose of the development of the surface-modified nanoparticle-incorporated gel formulation (MPPs-gel) was to deliver the therapeutic drug to the targeted site for a prolonged systemic effect *via* intravaginal delivery. Thus, the permeation analysis of the MTF-MI bi-formulation MPPs suspension (sample B) and MTF-MI bi-formulation MPPs-gel (sample C) was determined and compared with the MI-MTF-loaded conventional gel (sample A) to authenticate this concept. The cumulative amount of MTF drug released was estimated to be 0.51 ± 0.04 mg cm^−2^ (sample A), 1.61 ± 0.05 mg cm^−2^ (sample B), and 1.32 ± 0.06 mg cm^−2^ (sample C). The cumulative amount of MI drug released was estimated to be 0.43 ± 0.02 mg cm^−2^ (sample A), 1.52 ± 0.2 mg cm^−2^ (sample B), and 1.34 ± 0.21 mg cm^−2^ (sample C), as shown in Fig. 9(c) and (d). The slope of the linear portion of the drug permeation profile and the *J*_ss_ value of the formulation are presented in Table 3. The steady-state flux (*J*_ss_) of MTF permeation through samples A, B, and C was found to be 79.62 ± 1.33 μg h^−1^ cm^−2^, 254.77 ± 1.39 μg h^−1^ cm^−2^ and 203.82 ± 1.17 μg h^−1^ cm^−2^, respectively. Similarly, the steady-state flux (*J*_ss_) of MI permeability through samples A, B and C was estimated to be 68.79 ± 0.69 μg h^−1^ cm^−2^, 240.45 ± 0.81 μg h^−1^ cm^−2^ and 2.8.92 ± 0.94 μg h^−1^ cm^−2^, respectively (Table 3).

**Table 3:** *Ex vivo* permeation data for metformin and myoinositol

Permeation studies	Metformin			Myoinositol		
	Conventional gel (sample A)	MPPs-suspension formulation (sample B)	MPPs-gel formulation (sample C)	Conventional gel (sample A)	MPPs-suspension formulation (sample B)	MPPs-gel formulation (sample C)
Steady-state flux (*J*_ss_) (μg h^−1^ cm^−2^)	79.62 ± 1.54	254.77 ± 5.78	203.82 ± 4.81	68.79 ± 2.49	240.45 ± 5.14	208.92 ± 3.98
Permeability coefficient (cm^2^ h^−1^)	3.98 ± 0.4	12.73 ± 0.8	10.19 ± 0.79	3.44 ± 0.12	12.02 ± 0.89	10.46 ± 0.27

The obtained results for sample B and sample C suggest that these formulations were deeply permeated with a higher concentration through the vaginal tissue of the animal compared to sample A. The improved permeation through the MPPs-gel was observed to be initially limited, which was enhanced considerably after 3 h compared to the MPPs suspension. Thus, the time taken by the gel in the MPPs-gel formulation was due to the structural changes in the mesh of the gel and its altered viscosity by the acidic pH of the vaginal cavity.^53^ Moreover, the formulation showed adequate permeation through the vaginal membrane across its inherent barrier. The increased permeation from MPPs and MPPs-gel was achieved due to the nano-size of the particles (≤200 nm) and the presence of neutral surface charge on the particles because in a limited charge condition, the repulsive and steric forces got reduced, which ultimately helped the permeation of the nanosized particles through the mucosal tissue. The carbomer gel successfully held the MPPs as its cargo in the vaginal cavity, thus reducing the drug loss/formulation loss.^55,56^ Consequently, the permeation through the conventional gel was slow with limited release during the entire study, which may be due to strong adhesive force and stearic hindrance. Hence, the drugs released from sample A were deposited on the epithelial layer of the tissue, leading to low permeability. Therefore, the intravaginal delivery of MPPs-gel corroborated the better prolonged release behavior compared to the conventional vaginal gel.

### 3.8 Confocal microscopic study

**Fig. 10:**
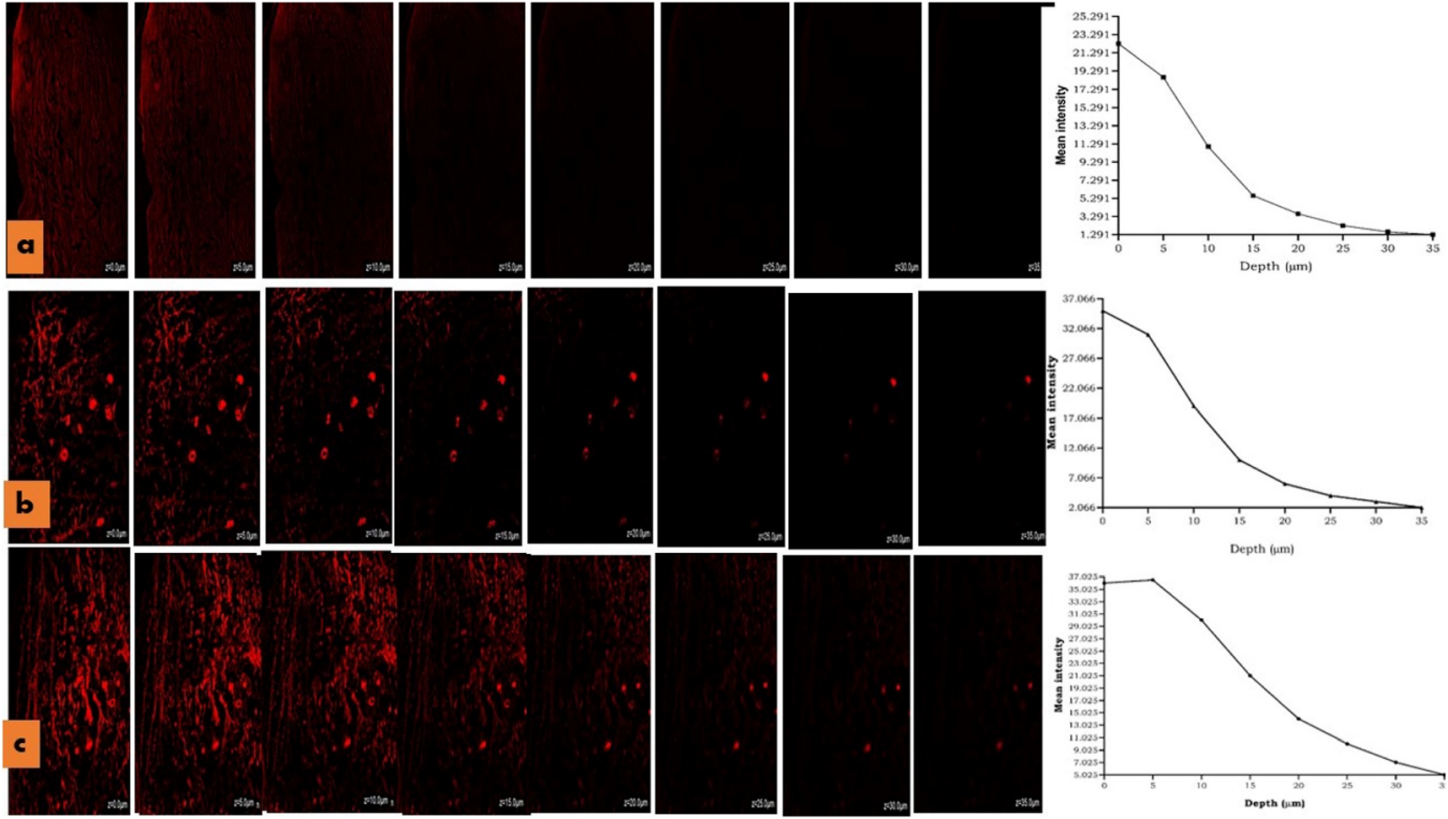
Confocal laser microscopy images showing vaginal membrane permeation of the rhodamine B-loaded conventional gel (a), rhodamine B-loaded MPPs (b) and rhodamine B-loaded MPPs-gel (c). The red colour indicates the fluorescence intensity.

For the intravaginal drug delivery system, the permeation of drug/MPPs across the mucosal vaginal barriers was demonstrated to be a critical factor, which should presumably maintain a minimum effective concentration of therapeutic drug at the ovarian tissue while being inserted through the uterovaginal pathway.^14^ The present study was conducted on the vaginal mucosa tissue of a goat to determine the penetration depth and intensity of MPPs and MPPs-gel, which were compared with the conventional gel. The prepared slides of the sample were analysed under a confocal microscope. The Rhodamine dye-loaded gel showed the highest intensity on the surface of the vaginal mucosa at ∼0 μm, whose intensity was reduced as the depth of the tissue increased and it became negligible at the depth of 10 μm. In contrast, the rhodamine-loaded MPPs exhibited high fluorescence intensity at 15 μm, and as the penetration trajectory in the vaginal tissue increased to 35 μm, their intensity diminished, while appearing to be more prominent in the blood vessels, as presented in Fig. 10. Thus, it can be inferred that the optimized formulation will sufficiently traverse and has significant ability to reach the ovary through the systemic circulation of blood vessels.^26,57^

### 3.9 Irritation studies on vaginal tissue

**Fig. 11:**
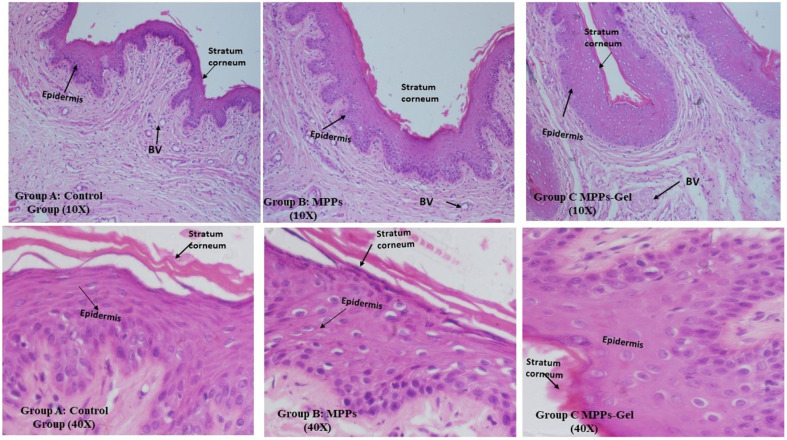
Histological investigation at 10× and 40× depicting the treatment effect on vaginal tissues after intravaginal administration of the optimized formulations *vs.* the control group.

The histological assessment of the excised vaginal tissue of the control group of animals showed that Group A (control group) depicted the normal structure of H & E stained histology of vaginal tissue. In this group, the mucosal layer appeared to be intact, having stratified squamous tissue of epithelium together with connective tissues of sub-epithelium, which was comprised of blood vessels without any inflammatory cells, as shown in Fig. 11 (Group A).

The stratified squamous epithelium seemed to have a normal thickness compared to the control group. Also, the basal layer and sub-mucosa layer of connective tissue were intact and in similar condition. There was no sign of sub-epithelial inflammatory reaction and hemorrhagic lesions. Thus, the formulated MPPs and MPPs-gel did not cause any inflammation or irritation after intravaginal drug delivery.^10,58^ All these findings can clearly be seen in Fig. 11 (Group B and C) at 10× and 40×.

### 3.10 *In vivo* animal studies

The estrous cycle is the reproductive cycle of female rats, which is considered to be identical in reproductive activities to the human menstrual cycle. Its different phases can be analysed by the cell types, as seen in the vaginal smears. The phases, *i.e.*, proestrus, estrous, metestrus, and dioestrus phases, are functionally corelated with the menstrual cycle phases. The PCOS model of animals was investigated in the PC group (group B) and compared with the rats of the negative control group (group A). The microscopic examination of the vaginal smears collected from the animals of group B for the estrous cycle assessment indicated a continuous dioestrus phase, as primarily indicated by the nucleated epithelial cells and their predominance during the two-week study of compared to the animals in group A. The obtained data was validated by the related graph plots. The PCOS group displayed insulin resistance, as revealed by the significantly enhanced fasting insulin level compared to the healthy control group (^###^*P* < 0.001), as shown in Table 4. Furthermore, the testosterone, estradiol, and luteinizing hormone levels were enhanced and the FSH level was reduced with a high significant value (^###^*P* < 0.001) compared with group A (NC), as observed in the extended data of Table 4. Thus, the disrupted estrous cycle, ovarian morphology/weight, insulin, and hormone level authenticated the PCOS-like conditions in the animals of the PC group compared with the healthy rats (group A), as reported in previous research.^59,60^

**Table 4:** Estimated biochemical parameters for PCOS treatment

Parameters	NC (Group A)	PC (Group B)	Gel (Group C)	MPPs-gel (Group D)
Serum insulin (uIU mL^−1^)	13.5 ± 0.84	18.21 ± 0.95^a^	16.98 ± 1.03^b^	15.91 ± 0.99^b^
Testosterone (ng mL^−1^)	0.40 ± 0.02	1.01 ± 0.03^a^	0.63 ± 0.01^b^	0.52 ± 0.04^b^
Luteinizing hormone (mIU mL^−1^)	3.12 ± 0.58	8.41 ± 1.04^a^	6.51 ± 0.8^b^	5.19 ± 0.68^b^
Follicle stimulating hormone (mIU mL^−1^)	6.23 ± 0.56	3.57 ± 0.82^a^	4.51 ± 0.69^b^	5.13 ± 0.71^b^
Estradiol (pg mL^−1^)	49.97 ± 1.91	64.06 ± 1.18^a^	62.14 ± 1.68^b^	59.82 ± 1.45^b^

a Statistics are expressed as mean ± SD (*n* = 6). One-way ANOVA together with Tukey's multiple comparisons test was used. In contrast to the negative control group and control groups: ^###^*p* < 0.001.

b In contrast to the positive control group: ***p < 0.001, **p < 0.01, *p < 0.05.

**Fig. 12:**
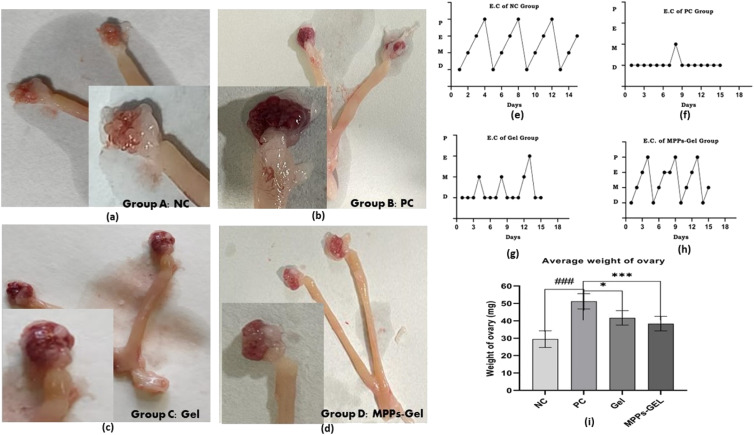
Comparative images: (a) ovary of the healthy animals of control group (Group A); (b) cystic condition of the ovary of the animals in the PC control group (Group B); (c) ovary revealing the treatment effect of the conventional vaginal gel on the animals in the gel group (Group C) and (d) ovary revealing the treatment effect of MPPs-gel on the animals of the MPPs-gel group (Group D). The other side shows the graph of the estrous cycle in the different groups: (e) estrous cycle (EC) of the control group of healthy animals; (f) EC of the PCOS model of the control group of animals (Group B) and (g and h) EC of treatment gel and MPPs-gel group, respectively. (i) Change in the average ovary weight of the respective groups .

Fig. 12(a)–(d) represent the morphological condition of the ovaries. The photograph of the control group of the PCOS model (Group B) showed a large bulb-like heavy ovary compared to the ovary of the healthy control animal (Group A). Alternatively, the post-treatment effect of the MPPs-gel formulation on the ovarian morphological condition seemed to be improved compared to the gel treatment group and control PC group of animals. The visualization authenticated the amelioration of PCOS by the treatment with the proposed formulation.

The post-treatment effect of the MPPs-gel formulation on the estrous cycle determined by vaginal smear of the animals revealed that all the phases of the estrous cycle were observed compared to the control and group C. The regular estrous cycle of the MPPs-gel group (group D) was corroborated by the presence of all stages of the estrous cycle, as shown in Fig. 12(e)–(h). The obtained data authenticated the recovery of the reproductive system, while eliciting improved ovarian function, ovulation, and follicular development compared to group B and group C. The images and accompanying data of average ovary weight variations in the treatment groups compared to the control group corroborate these findings, as given in Fig. 12(i). In contrast, the animals in group C (conventional gel treatment group) did not show a regular estrous cycle, given that the graph indicated an irregularity in their cycle. The obtained results for group D (MPPs-gel formulation) were consistent with the reported studies.^61^

In the diagnosis of insulin receptor sensitivity, the fasting insulin level is considered to be an essential parameter given that the molecular binding of insulin with the receptors is related to the serum insulin level. The obtained data of the serum insulin level of the MPPs-gel group suggested a high significant value (***P < 0.001) compared to the control group (group B). In the case of the conventional gel-treated animals (group C), no statistical variation (*P* > 0.05) was observed compared with the control (group B), as mentioned in the Table 4. Our results are consistent with other reported outcomes.^62^

Testosterone is the male sex hormone that regulates the male characters. However, it is produced in limited amounts in the female ovaries as needed for estradiol production. The luteinizing hormone produced by the pituitary gland plays an essential role in ovulation, followed by triggering estrogen and progesterone production. The excessive production of androgen due to the high binding capacity of free insulin with luteinizing hormone (hyperandrogenism) is responsible for the development of cystic follicles due to follicular arrest, inducing PCOS. The post-treatment effect of MPPs-gel showed significantly reduced (***P < 0.0005) levels of luteinizing hormone, testosterone, and estrogen levels and improved follicle-stimulating hormone (FSH) level with high statistical variation (***P < 0.0002) in comparison to the control group (group B). Similarly, in the case of the post-treatment therapeutic effect of the conventional gel (group C), it revealed a statistically less significant variation (*P < 0.01) (Table 4). Thus, the obtained data authenticated the findings reported by.^63,64^

The therapeutic effect of the proposed MPPs-gel formulation revealed a promising delivery system that significantly reached the targeted area and ameliorated the PCOS in the animal model. Alternatively, the vaginal administration of the conventional gel formulation may not yield the desirable therapeutic outcomes given that it would not fully reach the targeted area. Moreover, it can be concluded that the conventional gel formulation only adheres superficially to the mucosal lining and does not penetrate deeply in the blood circulation.

## 4. Conclusion

The bi-formulation MPPs-gel as a carrier of MTF and MI was developed for intravaginal drug delivery and exhibited a synergistic effect, where it could cross the vaginal barrier for systemic targeted delivery of the selected therapeutic compounds, as acknowledged by the outcomes of the *ex vivo* release studies and confocal microscopy. The systematically designed bi-formulation (due to quantitative high dose of the therapeutically active drug) with potentiated entrapment efficiency led to a high payload and its effective neutral surface charge endowed it with high mucosal penetration ability to reach the targeted ovaries. The post-treatment findings of the MPPs-gel in the *in vivo* studies facilitated a regular estrous cycle, as validated by the identification of different cells of the corresponding phases of the cycle compared to the control group. The biochemical estimation displayed the excellent pharmacological effects of MPPs-gel compared to the control and conventional vaginal gel treatment. Thus, the success of the proposed formulation was firmly validated by its ameliorative effect on the appropriate PCOS animal model. Furthermore, the pharmaco-technical suitability constructed a pharmaceutical product with good quality attributes and expected safety and therapeutic effect of the optimized formulation. However, although its purported success has been evaluated in a suitable PCOS animal model to some extent, the final therapeutic outcomes can only be assessed by clinical studies.

## Abbreviations

PCOS: Polycystic ovarian syndrome
QbD: Quality by design
MI: Myoinositol
MTF: Metformin
DCI: d-Chiro-inositol
PLGA: Poly-lactic-*co*-glycolic acid
MI-MPPs: Myoinositol-loaded mucus penetrating particles
MTF-MPPs: Metformin-loaded mucus penetrating particles
QTPP: Quality target product profile
QoL: Quality of life
RA: Risk assessment
RAM: Risk assessment matrix
MAs: Material attributes
FMEA: Failure mode effect analysis
CMAs: Critical material attributes
CPPs: Critical process parameters
CQA: Critical quality attributes
CCD: Central composite design
MPPs-gel: MTF-MI bi-formulation MPPs incorporated gel
ARRIVE: Animal research reporting of *in vivo* experiments
TEM: Transmission electron microscopy
DSC: Differential scanning calorimeter
LCMS: Liquid chromatography-mass spectrometry
FDC: Franz diffusion cell
SVF: Simulated vaginal fluid
D1: Drug 1 (metformin)
D2: Drug 2 (myoinositol)
EE: Entrapment efficiency

## Authors contribution

Uzma Farooq (U. F.): methodology, writing-original draft, writing-review and editing; Abdullah Alshetaili (A. A.): data curation; Mohd Aamir Mirza (M. A. M.): data curation; Sradhanjali Mohapatra (S. M.): software and validation; Pooja Jain (P. J.): validation; Nazia Hasan (N. H.): software; Zeenat Iqbal (Z. I.): project administration; writing-review and editing; and Asgar Ali (A. A.): project administration, writing-review and editing. All authors have read and agreed to the published version of the manuscript.

## Conflicts of interest

The authors declare no conflict of interest.

## Supplementary Material

NA-006-D3NA00943B-s001
