# Supplementary material for: In silico and in vitro assessment of an optimized QbD-guided myoinositol and metformin-loaded mucus-penetrating particle-based gel for the amelioration of PCOS
Source: Nanoscale Adv. 2024 Jan 3;6(2):648–68. doi: 10.1039/d3na00943b (PMC10791119; doi:10.1039/d3na00943b)
Supplement: NA-006-D3NA00943B-s001 [file NA-006-D3NA00943B-s001.pdf]

Electronic Supplementary Material (ESI) for Nanoscale Advances.
This journal is © The Royal Society of Chemistry 2023

SUPPLIMENTARY MATERIAL

Table S1: The quality target product profile (QTPPs) elements of the nano-appended formulation (MPPs) along with CQAs and their related justification

QTPP elements	Target	CQA	Justification
Clinical purpose (Therapeutic effect)	Reach the ovarian tissue (Both hydrophilic APIs (MF and MI, the potential target to normalize the endocrine hormones and treat the PCOS	-	Both of these drugs are reported to come under the first-line treatment for PCOS
Proposed pharmaceutical formulation	Surface-modified nanoparticles (MPPs)-gel	-	To improve therapeutic efficacy and patient compliance
Drug delivery system (route of administration)	Intra-vaginal drug delivery system	-	Deeply penetrate through the vaginal tissue to reach the systemic circulation through the uterovaginal pathways
Vaginal tolerability (irritation)	No irritation<br>No any allergic effect<br>No inflammation	-	Dermal effect
Targeted area	Ovaries	-	Effect on therapeutic efficacy and help to ameliorate PCOS
Dosage form strength	Good	-	Influence the frequency of dosing and dose
Particle diameter	≤200nm (small)	Yes	Effect on MPPs penetrability, targeted delivery, uniform distribution, and drug release
Polydispersity index	≤0.3 (uniform)	Yes	Effect on MPPs penetrability, targeted delivery, uniform distribution, and drug release
ζ potential	More neutral than -10mV	Yes	Effect on penetrability into the mucus layer, uniform distribution, and retention time
Biodegradable polymer concentration	Optimized	Yes	Facilitate particle size/shape, PDI, drug entrapment, and ζ potential
Surfactant concentration	Optimized	Yes	Effect on formulation development, particle size, and shape.
Carbomer concentration	Optimum	Yes	Effect on texture and viscosity of MPPs-gel formulation
Glycerol	Optimum	Yes	Moisturizing effect on vaginal tissue
Lactic acid	Vaginal pH	Yes	maintain suitable mucosal environment
pH	Vaginal pH	Yes	Effect on vaginal mucus membrane irritation and inflammation of the tissue
Temperature	50-60ºC	Yes	Impact on nano-formulation development

Drug entrapment efficiency	High	Yes	Facilitate the dosing quantity and drug delivery system
Drug release	Sustained	Yes	Facilitate the dosing quantity and drug delivery system
Needle type	Small	Yes	Effect on particle size and PDI
Injection rate	Optimized	Yes	Effect on particle size and PDI
Stirring speed	High	Yes	Effect on particle size, PDI, and stability
Stirring time	Optimized	Yes	Effect on particle size, PDI, and stability

CQAs	Clinical objective	Route of administration	Dosage form	Method	Vaginal tissue irritation	Retention time	Stability	Dosage form applicator
PS	Medium	Medium	High	High	Medium	Medium	High	Low
PDI	Low	Low	High	High	Low	Low	High	Low
ZP	Medium	High	High	Medium	Low	Medium	High	Low
EE	Low	Medium	High	High	Low	High	High	Low
TA	Low	Low	High	Low	Medium	Medium	Medium	High
RS	Low	Low	Medium	Low	Medium	Medium	Medium	High
pH	Medium	Medium	Medium	Low	High	Medium	Medium	Low
Release	High	High	High	Medium	Low	High	High	Medium

Figure S1: The RAM facilitated the QAs Vs QTPPs relationship

QAs	MAs										PPs					
	PLGA grade	PLGA conc.	Organic solvent	Drug conc.	PF 127 conc.	Carbomer grade	Carbomer conc.	TEA	Glycerine	Lactic acid	Injection rate	Injection speed	Temperature of MPPs	Needle size	Stirring time of mpps	Stirring time of carbomer solution
PS	Low	High	Medium	Medium	High	Low	Low	Low	Low	Low	High	High	High	High	Medium	Low
PDI	Low	High	Medium	high	High	Low	Low	Low	Low	Low	High	High	High	High	Medium	Low
ZP	Medium	Medium	Low	High	High	Medium	Medium	Medium	Medium	Medium	Low	Low	Low	Low	Low	Low
EE	Medium	High	Low	High	High	Low	Low	Low	Low	Low	Medium	Low	Medium	Low	Medium	Low
TA	Low	Low	Low	Low	Low	Medium	High	Medium	Medium	Low	Low	Low	Low	Low	Low	Medium
RS	Medium	Medium	Low	Medium	Medium	Medium	High	Medium	Medium	Medium	Medium	Low	Low	Low	Low	Medium
pH	Low	Medium	Low	Low	Medium	High	High	Medium	Medium	High	Low	Low	Low	Low	Low	Low
Release	High	Medium	Low	Medium	Medium	Medium	Medium	Medium	Low	Medium	Medium	Medium	Medium	Medium	Medium	Medium

Figure S2: The RAM facilitated the QAs Vs MAs/PPs relationship

Table S2: FMEA of different risk factors involved in development of formulation which also include their related failure mode, potential cause and their control. The severity score (S), probability of occurrence (O) and detectability (D) scores of MAs and PPs in relation with their failure mode were revealed. The RPN number of risk variable were calculated and classified accordingly into high, medium and low risk.

**Risk variable**	**Failure mode**	**Failure effect**	**S**	**Potential cause**	**O**	**control**	**D**	**RPN**	**Risk**
**QAs**									
**Particle size**	Large particle size (>200nm)	Failure to deep mucosal penetration, reach the targeted area and dose uniformity issue	9	Change in concentration of polymer and surfactant<br>Change in temperature, stirring speed and time	8	The optimum and precise quantity of polymer and surfactant will significantly affect the particle along with the control/constant temperature, stirring speed and time	9	512	High
**PDI**	>0.3	Alter in dose uniformity, uniform distribution of drug in the particles, and drug loading capacity	8	Major factors are Change in concentration of polymer and surfactant, change in temperature, stirring speed and tme	8	The optimum and precise quantity of polymer and surfactant will be significantly affecting the particle along with the control/constant temperature, stirring speed and time.	8	512	High
**Zeta potential**	Highly negative or highly positive surface charge	failure to cross the vaginal mucosal membrane/barrier and not capable to reach on the targeted area<br>Failure to maintain MIC level in a targeted area<br>Stability issue of formulation	7	Ionic property of selected materials (especially those material which are present on the outer shell of particles) are key factor to affect the zeta potential	7	The selected coating material and surfactant are potent to neutralize nanoparticle surface charge.	6	294	Medium

**Drug entrapment efficiency**	A low percentage of drug entrapment efficiency	Alter in dose uniformity, uniform distribution of a drug in the particles, and minimum effective concentration (MIC)	8	Formulation development method, surfactant concentration and temperature are key factor	8	Method should be developed based on drug solubility profile and log P value<br>The optimum amount of surfactant affects the drug entrapment efficiency	7	448	High
**Drug loading**	Low % of drug loading	Alter in dose uniformity, uniform distribution of a drug in the particles, and minimum effective concentration (MIC)<br>Negative impact on dose capacity	6	Formulation development method, surfactant concentration and temperature are key factor	7	Method should be developed on the basis of drug solubility profile and log P value<br>Optimum amount of surfactant affect the drug entrapment efficiency	7	294	Medium
**Drug release**	Low limits of drug entrapment efficiency /pharmacological action	Formulation failure as its effects on pharmacological effect	6	Drug entrapment efficiency and drug loading capacity	6	Maximum limit of drug entrapment efficiency, loading capacity and sustained release	7	294	Medium
**Content uniformity**	Non-uniform drug	Negative impact on dose capacity and drug release	5	High PDI, and inadequate temperature range	5	Optimum PDI, temperature and stirring speed and time	4	100	Low
**MAs**									

**Organic solvent concentration**	Inadequate quantity	Impact on formulation development and emulsification	3	Inadequate quantity affects the formulation development	2	Optimum quantity helps in proper emulsion formation during the processing of formulation development	3	18	Low
**Polymer concentration**	Inadequate particle size, PDI, drug loading capacity	Drug release, drug loading capacity, entrapment efficiency, particles size, and PDI are the majorly affected factors	9	Polymer concentration majorly contributed to the formulation development that affects the drug release, drug loading capacity, entrapment efficiency, particles size, and PDI	8	an accurate and precise quantity of polymeric concentration possessed optimum results	9	684	High
**PF127 concentration**	Inadequate particle size, PDI, drug loading capacity and stability	Drug loading capacity, entrapment efficiency, stability, particles size, and PDI are affected by surfactant concentration	8	High surfactant concentration gave better results of particle size/PDI but low drug entrapment efficiency/loading capacity while excessive low concentration facilitates large particle size and high PDI	8	Optimum concentration facilitates required particle size/PDI and a high % of drug entrapment efficiency/loading capacity. It also provides stability to the formulation	8	512	High

**Drug concentration**	Low drug loading capacity	High concentration might affect on the quality of particle size and low entrapment efficiency. Excessive low concentration fail to meet the therapeutic effect of drug	8	High concentration might affect the quality of particle size and low entrapment efficiency	8	The optimum concentration provides better therapeutic effect and improved the quality of the product	8	512	High
**Formulation method**	The solvent evaporation method failed to load hydrophilic drugs	Less % of drug entrapment efficiency and loading capacity	5	An alternative method might improve the entrapment efficiency and loading capacity	7	The double emulsion method provided improved drug loading capacity and entrapment efficiency	7	245	Medium
**Caromer concentration**	Either very high viscosity or very low	Solidified/ liquified gel might effect on texture and viscosity of gel while less viscose gel might be expelled from the vaginal cavity thus effect on the therapeutic dose. It also effects on patient compliance.	8	A change in concentration could alter the texture and viscosity of the gel	6	Optimum concentration could improve the texture and viscosity of gel	6	288	Medium

**Glycerol**	High concentration	Might affect on physical properties of gel	6	High lubricating effect on vaginal cavity	5	Optimum concentration could moisturize the vaginal tissue	6	180	Medium
**Lactic acid**	High concentration	Alter pH of vaginal cavity	6	High concentration might cause burning sensation/irritation	5	Optimum comcentration maintain vaginal pH.	6	180	Medium
**pH**	Outside the limit of the simulated vaginal pH range and skin irritation	Skin irritation and change in release profile	6	Change in concentration of required component	6	Optimum pH needs to avoid irritation, inflammation, and discomfort on the vaginal area	5	180	Medium
**Log P**	Reduce permeability	Change in permeability	2	Physicochemical properties of API	3	-	3	18	Low
**PPs**									
**Temperature**	Inadequate temperature range	Inadequate formulation development, change in particle size and PDI affect drug entrapment efficiency, loading capacity and uniformity	7	At low temperatures, failure of emulsification in the processing of nanoparticles, enhanced particle size, and PDI	7	Optimum constant temperature	6	294	Medium

**Needle type**	High range of particles size	Alteration in particle size and PDI of the formulation	2	Change in needle size	3	Proper screening of needle size	2	12	Low
**Injection rate**	High range of particles size	Enhance particle size and PDI of the formulation	4	Non-uniform plunger pressure leads to change the injection rate	5	Constant plunger pressure and optimum injection rate	4	80	Low
**Stirring speed**	High range of particles size and increase in PDI	An extended range of particles size and high PDI are responsible for non-uniform dose, failure to cross the vaginal mucosal membrane/barrier, also effect on drug entrapment efficiency	6	Slow and irregular stirring speed	7	High and constant stirring speed	6	252	Medium
**Stirring time**	Instability		7		7		6	294	
**homogeneity**	Non-uniform drug distribution and dose		3		2		3	18	

Table S3 (Supplementary material): Total 19 runs of formulation trials with the response of CQAs

	**MAs**		**PPs**					
**Trials**	**PLGA polymer**	**PF127(%)**	**Stirring speed**	**S. time**	**Temperature (℃)**	**Inj. Rate (ml/min)**	**PS**	**PDI**
F1	**10**	0.4	1500	6h	60	1	125.6	0.156
F2	**20**	0.4	1500	6h	60	1	167.1	0.178
F3	**30**	0.4	1500	6h	60	1	197.25	0.169
F4	40	0.4	1500	6	60	1	251.41	0.245
F5	30	**0.1**	1500	6	60	1	210.12	0.412
F6	30	**0.5**	1500	6	60	1	195.15	0.157
F7	30	**1.0**	1500	6	60	1	139.87	0.0782
F8	30	0.4	**500**	6	60	1	248.69	0.314
F9	30	0.4	**1000**	6	60	1	221.09	0.287
F10	30	0.4	**1500**	6	60	1	198.47	0.192
F11	30	0.4	1500	**3**	60	1	215.89	0.478
F12	30	0.4	1500	**5**	60	1	196.78	0.287
F13	30	0.4	1500	**7**	60	1	198.47	0.194
F14	30	0.4	1500	6	**30**	1	578	1.083
F15	30	0.4	1500	6	**40**	1	201.98	0.277
F16	30	0.4	1500	6	**60**	1	199.44	0.178
F17	30	0.4	1500	6	60	**1**	184.28	0.217

F18	30	0.4	1500	6	60	**2**	238.86	0.374
F19	30	0.4	1500	6	60	**3**	289.78	0.318

Table S4 (Supplementary material): Optimization range of some PPs

S.no.	Process parameters (PPs)	Optimized value
1.	Temperature (ºC)	60°C
2.	Stirring speed (rpm)	1500rpm
3.	Stirring time (hrs)	6-8hrs
4.	Injection rate (ml/min)	1ml/min

Table S5 (Supplementary material): Various factors and their level of the different ranges including axial points were applied in the DoE.

**Independent variable, CMAs**	**Level**				
	**-6.82**	**-1**	**0**	**+1**	**6.82**
**X1 = PLGA conc (mg)**	13.18	20.0	30.0	40.0	46.82
**X2 = PF127 conc(%)**	0.0636	0.20	0.30	0.40	0.7364
**X3 = Drug conc (mg) D1 or D2**	33.18	40	50.0	60.0	66.82

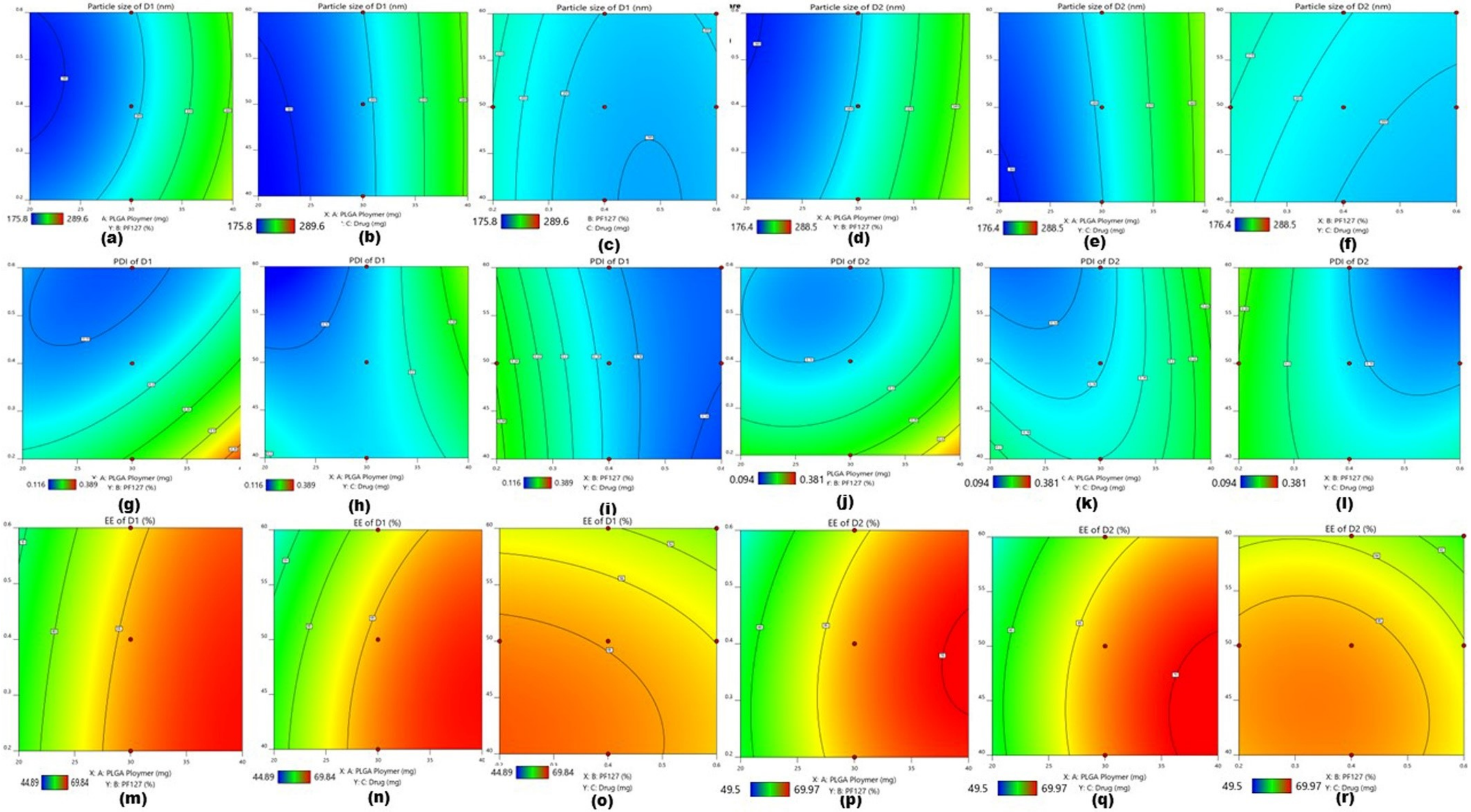

Particle size of D1 (nm)
175.8
289.6
X: A: PLGA Ploymer (mg)
Y: B: PF127 (%)
(a)
Particle size of D1 (nm)
175.8
289.6
X: A: PLGA Ploymer (mg)
Y: C: Drug (mg)
(b)
Particle size of D1 (nm)
175.8
289.6
X: B: PF127 (%)
Y: C: Drug (mg)
(c)
Particle size of D2 (nm)
176.4
288.5
X: A: PLGA Ploymer (mg)
Y: B: PF127 (%)
(d)
Particle size of D2 (nm)
176.4
288.5
X: A: PLGA Ploymer (mg)
Y: C: Drug (mg)
(e)
Particle size of D2 (nm)
176.4
288.5
X: B: PF127 (%)
Y: C: Drug (mg)
(f)
PDI of D1
0.116
0.389
X: A: PLGA Ploymer (mg)
Y: B: PF127 (%)
(g)
PDI of D1
0.116
0.389
X: A: PLGA Ploymer (mg)
Y: C: Drug (mg)
(h)
PDI of D1
0.116
0.389
X: B: PF127 (%)
Y: C: Drug (mg)
(i)
PDI of D2
0.094
0.381
PLGA Ploymer (mg)
B: PF127 (%)
(j)
PDI of D2
0.094
0.381
A: PLGA Ploymer (mg)
Y: C: Drug (mg)
(k)
PDI of D2
0.094
0.381
X: B: PF127 (%)
Y: C: Drug (mg)
(l)
EE of D1 (%)
44.89
69.84
X: A: PLGA Ploymer (mg)
Y: B: PF127 (%)
(m)
EE of D1 (%)
44.89
69.84
X: A: PLGA Ploymer (mg)
Y: C: Drug (mg)
(n)
EE of D1 (%)
44.89
69.84
X: B: PF127 (%)
Y: C: Drug (mg)
(o)
EE of D2 (%)
49.5
69.97
X: A: PLGA Ploymer (mg)
Y: B: PF127 (%)
(p)
EE of D2 (%)
49.5
69.97
X: A: PLGA Ploymer (mg)
Y: C: Drug (mg)
(q)
EE of D2 (%)
49.5
69.97
X: B: PF127 (%)
Y: C: Drug (mg)
(r)

Figure S3: Gontour plot of optimization of formulation represented the effect of independent variables on dependent variables. Here, graph a, c, e, g, i, k, m, o and q represented the contour plots of MTF-MPPs while graph b, d, f, h, j, l, n, p, and r represented the contour plots of MI-MPPs formulation

Table S6: Summery of fit on all the responses of respective drug having regression analysis between adjusted value vs predicted value

**CQAs**	**Model**	**Summary of fit**				
		**P-value**	**R^2**	**Adjusted R^2**	**Predicted R^2**	**Adequate precision**
**PS of MTF or D1(Y1)**	Quadratic	0.0001	0.9789	0.9599	0.9162	25.5939
**PS of MI or D2(Y2)**	Quadratic	0.0020	0.9652	0.9339	0.8644	20.8877
**PDI of D1(Y3)**	Quadratic	<0.0001	0.9983	0.9968	0.9897	83.4389
**PDI of D2(Y4)**	Quadratic	<0.0001	0.9952	0.9909	0.9831	53.3576
**EE of D1(Y5)**	Quadratic	<0.0001	0.9836	0.9688	0.8966	28.3225
**EE of D2(Y6)**	Quadratic	<0.0001	0.9927	0.9862	0.9642	41.1738

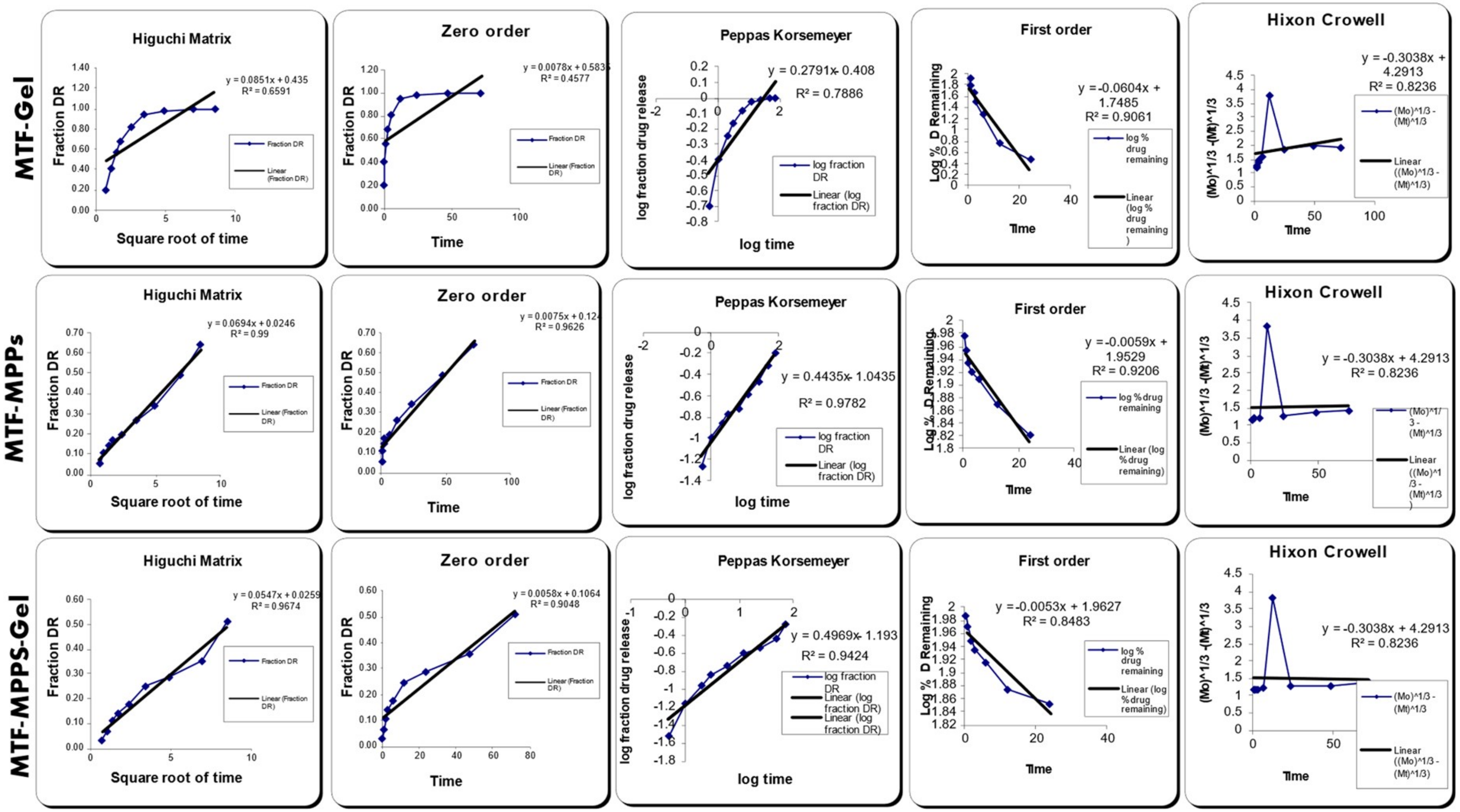

Figure S4: represents the kinetic model of the in-vitro release study: Sample A (MTF-Gel) follow first-order kinetic; sample B(MTF-MPPS) follows zero-order kinetic and sample C (MTF-MPPs-Gel): follows zero-order kinetic

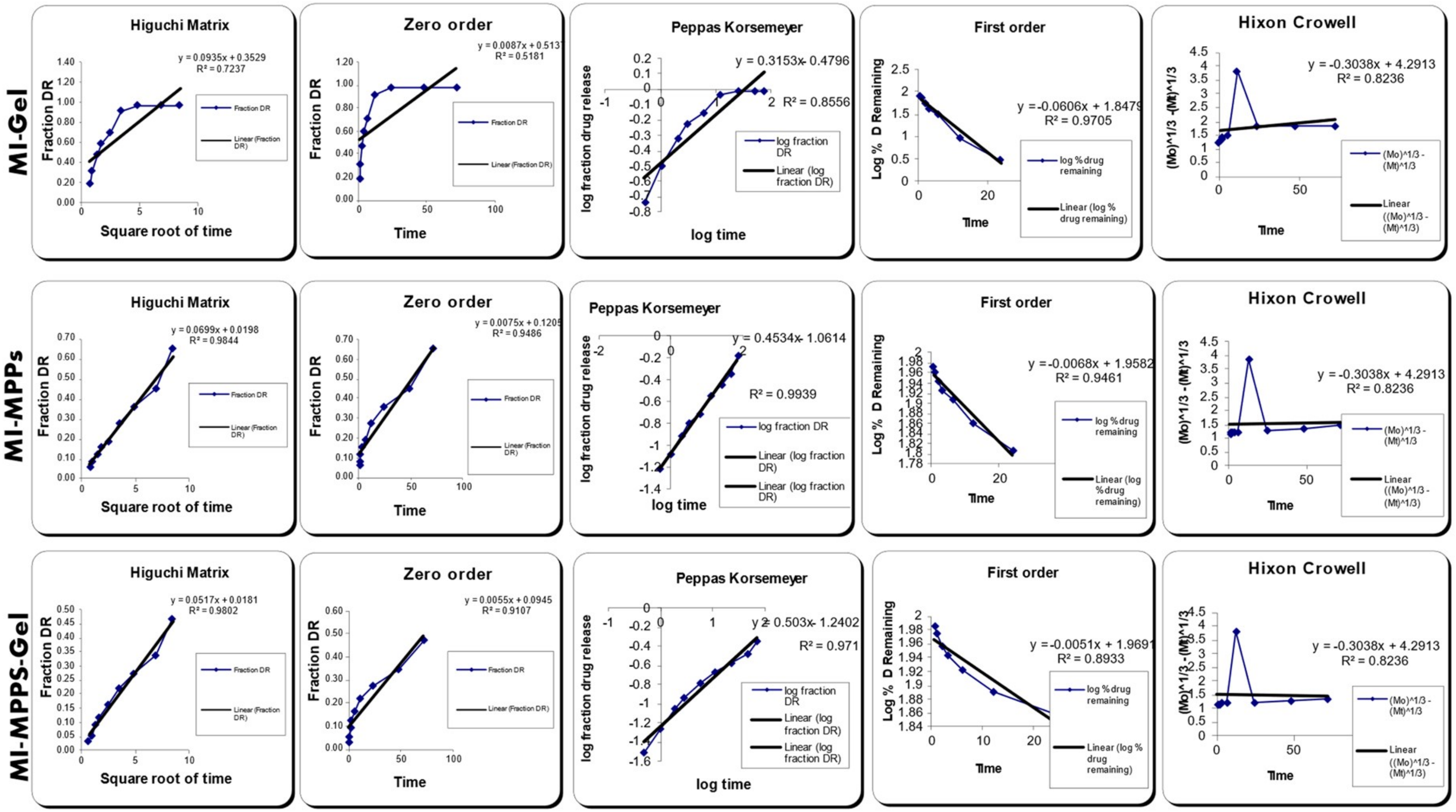

Figure S5: represents the kinetic model of the in-vitro release study: Sample A (MI-Gel) follows first-order kinetic; sample B(MI-MPPS) follows zero-order kinetics and sample C (MI-MPPs-Gel): follows zero-order kinetic
